# Arginase-2 enhances monocyte-endothelial interaction through regulation of integrin and membrane receptor expression: role in atherogenesis

**DOI:** 10.1186/s12929-026-01278-3

**Published:** 2026-07-13

**Authors:** Guillaume Ajalbert, Yuejun Yao, Sophie Rohrer, Matteo Caretti, Santhoshkumar Sundaramoorthy, Anastasia Rigkou, Michael Stumpe, Xiu-Fen Ming, Duilio M. Potenza, Zhihong Yang

**Affiliations:** 1 Laboratory of Cardiovascular and Aging Research, Department of Endocrinology, Metabolism, and Cardiovascular System, Faculty of Science and Medicine, University of Fribourg, Chemin du Musee 5, 1700 Fribourg, Switzerland; 2 Department of Biology, University of Fribourg, Chemin du Musee 10, CH-1700 Fribourg, Switzerland

**Keywords:** ARGINASE 2, Endothelial cells, Integrins, Inflammation, Monocytes

## Abstract

**Background:**

Circulating monocyte trans-endothelial migration is critical for vascular diseases. The monocyte surface proteins, i.e., integrins and membrane receptors are central in this process. Previous studies demonstrate that ablation of the mitochondrial *arginase-2* (*ARG2*) reduces monocyte/macrophage infiltration in cardiovascular disease. We further investigate whether ARG2 regulates integrin and surface receptor levels in monocytes and facilitates trans-endothelial migration, contributing to atherogenesis.

**Methods:**

For this purpose, human THP1 cells deficient in *ARG2* gene (THP1^*ARG2–/–*^) were generated by CRISPR-U^™^-mediated genome engineering. Atherosclerotic *Apoe*^*–/–*^*Arg2*^+*/*+^ mice and *Apoe*^*–/–*^*Arg2*^*–/–*^ mouse models are used. Proteomic profiling, cellular/molecular biology methods, and confocal microscopy were utilized for gene or protein expression analysis.

**Results:**

As compared to the control THP1^*WT*^ cells, the THP1^*ARG2–/–*^ cells reveal decreased adhesion and trans-endothelial migratory activities towards chemoattractants in a transwell co-culture system, accompanied by lower levels of αL and α4 integrins, CD99 and PECAM1 (CD31) (surface molecules for trans-endothelial activity) and CCR2 (the chemoattractant receptor). The monocyte adhesion to endothelial cells is reduced by blocking LFA-1 (αLβ2) or VLA-4 (α4β1) integrin. Pro-inflammatory polarization of the THP1 cells with LPS does not affect the integrin and surface receptor levels, however, enhances release of several pro-inflammatory cytokines, which is reduced in THP1^*ARG2–/–*^ cells due to reduced TLR4–ERK–NF-κB signaling. Conditioned medium from the LPS-primed THP1^*WT*^ reveals higher capacity to enhance endothelial VCAM-1 and ICAM-1 levels than the THP1^*ARG2–/–*^ cells, that is partly mediated by IL-1β. Moreover, ARG2-dependent TGF-β signaling was found to selectively regulate αL expression in monocytes. Finally, as compared to *Apoe*^*–/–*^*Arg2*^+*/*+^ mice, the *ApoE*^*–/–*^*Arg2*^*–/–*^ mice show significantly decreased macrophage integrin, chemoattractant receptor levels, and atherosclerosis.

**Conclusions:**

Our study identifies ARG2 as a key regulator of monocyte-mediated vascular inflammation and atherogenesis by controlling integrin, chemoattractant receptor expression, and pro-inflammatory cytokine release through TLR4-ERK-NFκB signaling, highlighting ARG2 as a potential therapeutic target for vascular and chronic inflammatory diseases.

**Supplementary Information:**

The online version contains supplementary material available at 10.1186/s12929-026-01278-3.

## Introduction

Circulating monocytes are from the innate immune system and play essential roles in immune responses, inflammation, and tissue degeneration and repair [1]. In response to tissue injury, inflammation, infection, and tumor microenvironment, monocytes in the blood stream are activated and interact with vascular endothelial cells through the process that includes tethering and rolling of the cells along the activated endothelium, firm adhesion and trans-endothelial migration, leading to infiltration into tissues, where they undergo phenotype adaptation to the microenvironment and trans-differentiation to macrophages [2]. In cooperation with tissue resident macrophages from embryonic precursors, infiltrated monocyte-derived macrophages are involved in tissue injury, repair, regeneration, and tumor progression [3]. These processes of the monocyte-endothelial cell interaction are mediated by specific surface molecules such as selectins which mediate initial tethering and rolling of monocytes along the activated endothelial cells [2]. The monocytes then interact with chemokines bound on the endothelial surface heparan sulphate proteoglycans, resulting in the activation of integrins such as LFA-1 (also known as αLβ2-integrin) and VLA-4 (α4β1-integrin), which interact with ICAM-1 and VCAM-1 of inflamed endothelial cells, respectively, to ensure monocyte adhesion to the vascular endothelium [4]. The monocytes then undergo morphological polarization with redistribution of chemokine receptors and activated integrins to the leading edge. Afterwards, monocytes migrate toward interendothelial junctions and then transmigrate into the underlying tissues [2] through interaction of monocytic integrins (LFA-1, VLA-4, Mac1) with endothelial JAMs (JAM-A, JAM-B, JAM-C), allowing monocyte transmigration through endothelial tight junctions [5]. Finally, PECAM-1 (CD31) and CD99 haemophilic engagement and endothelial retraction lead to monocyte extravasation [6]. Moreover, chemoattractant/chemokine receptors, i.e., CCR2, CCR5, and CX3CR1 are also involved in response to their respective ligands released by inflamed cells [7, 8]. The interactions of these receptors with the chemoattractant/chemokine ligands further facilitate the monocyte trans-endothelial migration and recruitment of the cells in the tissue, leading to development and progression of inflammatory diseases such as atherosclerosis [7].

Our previous studies demonstrate that the mitochondrial enzyme arginase type 2 (ARG2) plays a role in chronic inflammation in aging and age-associated diseases including atherosclerosis [9]. Indeed, *Arg2* deficiency in mice on *ApoE*^*–/–*^ background reduces macrophage inflammatory cytokine production, macrophage accumulation in the plaque, and reduces atherosclerotic plaque formation with a more stable plaque phenotype [9], demonstrating a role of *Arg2* in promoting inflammation and development of atherosclerosis. It is, however, not clear, how *Arg2* promotes macrophage accumulation in the vascular wall. Studies published in the literature suggest vital roles of monocyte/macrophage integrins in atherosclerotic plaque formation reviewed by Finney AC et al. [10]. Genetic deletion or blocking specific integrins by antibodies reduces macrophage recruitment and atherosclerosis in the *ApoE*^*–/–*^ mice [10–12].

In the current study, we therefore hypothesize that ARG2 may regulate integrin expression in monocytes/macrophages, which affects monocyte-endothelial interactions including adhesion and trans-endothelial migration, contributing to atherogenesis.

## Methods

### Reagents and antibodies

Cell culture reagents were purchased or obtained from the following sources: RPMI 1640 (RPMI-HXA) was from Capricorn scientific (Ebsdorfergrund, Germany). Penicillin/streptomycin (15140-122), fetal calf serum (FCS, A5256801), bovine serum albumin (15260-037) and glutamine (35050061) were from Gibco (Waltham, USA). Endothelial Cell Growth Medium Supplement Pack (C-392010) was from Promocell (Heidelberg, Germany). TNFα (cyt-223-b) was from Prospec (Rehovot, Israel). lipopolysaccharides (LPS, 44391) was from Merck (Darmstadt, Germany). BIRT-377 (4776), PD9849 (1213), IWP4 (5214) and IL-Ra (280-RA) were from R&D systems (Minneapolis, USA). MLN120B (32819) was from Cayman Chemical (Ann Arbor, USA). CFDA-SE (C1157) was from Invitrogen (Waltham, USA). ELISA kits for human TNFα (430204), human IL-6 (430504) and human IL-1β (437004) were from Biolegend (San Diego, USA). ELISA kit for human TGF-β (88–8350-88) was from Invitrogen (Waltham, USA). MTT cell proliferation assay kit (ab211091) was from Abcam (Cambridge, UK). Natalizumab solution (HY-108831A) was from MedChemExpress (New Jersey, USA). All catalog numbers and providers for primary and secondary antibodies are shown in Table 1.

**Table 1:** Antibodies and dilutions used for confocal microscopy and western blot

Antibody target	Dilution
ITGAL (sc-374172, Santa Cruz)	WB 1:750
ITGAL (TA332416, Origene)	IF 1:100
ITGA4 (sc-365569, Santa Cruz)	WB 1:750
ITGA4 (#8440S, Cell Signaling)	IF 1:100
ITGB1 (sc-374429, Santa Cruz)	WB 1:750
ITGB2 (sc-393790, Santa Cruz)	WB 1:750
ITGAM (sc-1186, Santa Cruz)	WB 1:750
ITGAM (#17800S, Cell Signaling)	IF 1:100
CCR2 (ab273050, Abcam)	IF 1:100
arginase 2 (#55003, Cell Signaling)	WB 1:1000
l-selectin (sc-390756, Santa Cruz)	WB 1:750
α-tubulin (T5168, Merck)	WB 1:4000
v-cam1 (ab134047, abcam)	WB 1:1000
i-cam1 (sc-8439, Santa Cruz)	WB 1:1000
JAM-1 (sc-53623, Santa Cruz)	WB 1:750
CD99 (sc-28389, Santa Cruz)	WB 1:750
CD31 (sc-376764, Santa Cruz)	WB 1:750
beta-actin (A5441, Merck)	WB 1:4000
NF-κB p65 (#6956, Cell Signaling)	WB 1:1000
phospho-NF-κB p65 (ser536) (#3033, Cell Signaling)	WB 1:1000
ERK (610123, BD biosciences)	WB 1:1000
Phospho-p44/42 MAPK (Erk1/2) (#9101, Cell Signaling)	WB 1:1000
Phospho-SMAD3 (sc-517575, Santa cruz)	WB 1:750
SMAD2/3 (#5678, Cell signaling)	WB 1:750
IBA1 (234308, Synaptic Systems)	IF 1:200
IRDye 800-conjugated affinity purified goat anti-rabbit IgG (9263221, BioConcept)	WB 1:5000
Alexa fluor 680-conjugated goat anti-mouse IgG (A-21057, Invitrogen)	WB 1:5000
Peroxidase AffiniPure^®^ Goat Anti-Rabbit IgG (H+L) (111-035-144, Jackson ImmunoResearch)	WB 1:5000
Peroxidase AffiniPure^®^ Goat Anti-Mouse IgG (H+L) (111-035-003, Jackson ImmunoResearch)	WB 1:5000
Alexa Fluor 488-cojugated goat anti-rabbit IgG (H+L) secondary Ab (A-11008, Thermo Fisher Scientific)	IF 1:400
Alexa Fluor 568-cojugated goat anti-rguinea pig IgG (H+L) secondary Ab (A-11075, Thermo Fisher Scientific)	IF 1:400

### Animal atherosclerotic models

The *Apoe*^–/–^*Arg2*^+/+^ mice from Jackson Laboratory and the generation of the *Apoe*^–/–^*Arg2*^–/–^ mice, both on C57BL/6 J background, as well as the animal experimental procedures were as previously described [9]. Briefly, to accelerate the atherosclerotic lesion formation, 10 weeks old male *Apoe*^–/–^*Arg2*^+/+^ and *Apoe*^–/–^*Arg2*^–/–^ mice were fed a high-cholesterol diet (HCD) for 10 weeks. At 20 weeks of age, animals were anesthetized with ketamine (100 mg/kg i.p.) and xylazine (10 mg/kg i.p.), the entire aorta from the heart to the iliac bifurcation were removed and collected. For immunostaining, the aortic roots (AR) were snap frozen in Optimal Cutting Temperature (OCT) compound and the 7 µm thick cryosections of the AR were prepared. Animal housing and experimentation were approved by the Service de la sécurité alimentaire et des affaires vétérinaires, Etat de Fribourg (2011_12_FR).

### Cell culture

Human umbilical vein endothelial cells (HUVEC) were cultured in RPMI-1640 (with 25 mmol/L HEPES and stable glutamine) containing 5% FCS and 1% penicillin/streptomycin supplemented with endothelial cell growth factor in porcine gelatin (1%) coated dishes. Before each experiment, cells were grown to confluence and cell cycle synchronized by culturing for 24 h in starvation medium (RPMI1640 with 25 mmol/L HEPES, stable glutamine, 0.5% FCS, 0.2% BSA and 1% penicillin/streptomycin). HUVECs were used at passages 3 to 4 for all experiments.

THP1 cells, a human monocyte-like cell line (ATCC, a monocyte cell line isolated from a one year’s old male), were cultured in RPMI (with 25 mmol/L HEPES and stable glutamine) containing 10% FCS and 1% penicillin/streptomycin. Cell density was maintained between 2 × 10^5^ and 1 × 10^6^ cells/mL. Before each experiment, cells were cell cycle synchronized by culturing them for 24 h in starvation medium (RPMI1640, 0.5% FCS, 0.2% BSA) at 5 × 10^5^ cells/mL.

*ARG2-*knockout human THP1 cell line (referred as THP^*ARG2–/–*^) was generated by CRISPR-UTM-mediated genome engineering with gRNA targeting exon 1 by Guangzhou Ubigene Biosciences Co., Ltd. The control THP1 cell line (referred as THP1^*WT*^) was generated with scramble gRNA and Cas9. *ARG2* gene or ARG2 protein deficiency was verified by sequencing and immunoblotting analysis. To compare the THP1^*WT*^ and THP^*ARG2–/–*^ cell proliferation and viability, THP1 cells were cultured for 4 days in starved medium (RPMI1640, 0.5% FCS, 0.2% BSA). Every 24 h, proliferation was measured using MTT assay and cell counting with trypan blue. Cell viability was also measured using trypan blue. The results show no significant effects of ARG2 deficiency on the THP1 cell proliferation and viability (Suppl. Figure 1).

For experiments that analyzed the effects of LPS, THP1 cells were cultured with LPS (0.1 µg/mL) for 24 h at the cell density of 5 × 10^5^ cells/mL. To prepare conditioned medium after LPS stimulation, cells were primed with LPS (0.1 µg/mL) for 24 h, then washed 3 times with PBS and kept in culture for 48 h in starvation medium. Then conditioned medium was filtered and stored at – 80  C for further experiments.

For experiments that analyzed the effects of NF-κB and ERK on integrin and cytokines expression. THP1 cells were cultured with LPS (0.1 µg/mL) for 24 h at the cell density of 5 × 10^5^ cells/mL with addition of a NF-κB inhibitor (MLN120B) at 20 µmol/L or an ERK inhibitor (PD98049) at 10 µmol/L. Then, cells were collected to perform further experiments.

For experiments who analyzed the effects of TGF-β1 on integrins. THP1 cells were cultured for 72 h with addition of a TGF-β receptor blocker (SB431542) at 10 µM. Then, cells were collected to perform further experiments.

### Proteomics

The THP1^*WT*^ and THP^*ARG2–/–*^ cells were starved (RPMI1640 + 0.5% FBS + 0.2% BSA + 1% penicillin/streptomycin) for 48 h. Cells were then washed 3 times with ice cold PBS and kept at − 80 °C. Then, the samples from the THP1^*WT*^ and THP^*ARG2–/–*^ cells in triplicates were analyzed by the Metabolomics and Proteomics Platform (MAPP) of the University of Fribourg, Switzerland) using a nanoLC-ESI–MS/MS systems (ThermoFisher) to obtain Proteomics profile of THP1. Results were expressed in log2 of all proteins. *p* < 0.05 is considered as statistically significant.

### Western blot

Cell lysate preparation, SDS-PAGE and immunoblotting, antibody incubation, and signal detection were performed as described previously [9]. Cells were first washed twice with PBS and lysed during 30 min under agitation at 4^◦^C in 1X RIPA lysis buffer supplemented with protease inhibitor cocktail (B14002, Sellckchem, Cologne, Germany) and phosphatase inhibitor cocktail (B15002, Sellckchem, Cologne, Germany). Homogenates were centrifuged at 13,000 × *g *for 15 min at 4 °C and supernatants were collected to determine protein concentration by Lowry method (500–0116, Bio-Rad, Hercules, USA). Equal amount of protein from each sample was heated at 95 °C for 15 min in loading buffer and separated by SDS-PAGE electrophoresis. Proteins were then transferred to PVDF membranes blocked with PBS-Tween-20 buffer supplemented with 5% skimmed milk. Membranes were first incubated overnight at 4 °C with gentle agitation in presence of the corresponding primary antibody. After three times washing with the blocking buffer, the membranes were then incubated two hours at room temperature with the corresponding anti-mouse or anti-rabbit secondary antibody. Signals were visualized using the Odyssey Infrared Imaging System (LI-COR Biosciences, Lincoln, USA), or the FUSION FX Imaging system (Witec AG, Luzern, Switzerland) for chemiluminescence, and quantified by Image Studio Lite (5.2, LI-COR Biosciences, Lincoln, USA). All antibodies and dilution are shown in Table 1**.**

### Adhesion assay under static condition

Adhesion assay in static condition was performed as described previously [9]. Briefly, for adhesion assays, the THP1 cells were labeled with CFDA-SE (5 μmol/L) in PBS at 37 °C for 8 min. The labeling was stopped by adding 1 mL of heat-inactivated FCS and incubated at room temperature for 1 min. The labeled monocytes THP1 were then added at a density of 37′500 cells/cm^2^ to the “activated” HUVEC monolayer (i.e., the endothelial cells were pre-treated with TNFα (10 ng/mL, 16 h) followed by three times of washing with cultured medium just before the experiments of monocyte adhesion assay (to avoid the influence of TNFα on monocytes). After incubation for 20 min at 37 °C, the non-adherent THP1 cells were washed twice with PBS and attached monocytes were fixed in 4% formalin. Images of adherent monocytes were acquired with a fluorescent microscope Zeiss Axio Observer A1 (three to four different fields per sample were captured). The number of adherent monocytes was counted using the Image J software.

For experiments in which LFA-1 activity was blocked, THP1 cells were treated with potent negative allosteric modulator of LFA-1 (BIRT-377) during 6 h at 20 µmol/L in starvation medium, then cell adhesion was assessed.

For experiments in which VLA-4 activity was blocked, THP1 cells were treated with humanized IgG4 neutralizing antibody (Natalizumab) that block the interaction of VLA-4 and VCAM-1, during 2 h at 15 µg/mL in starvation medium, then cell adhesion was assessed.

### Adhesion assay under shear stress

Adhesion assay in flow condition was performed as described previously [13]. Briefly, HUVECs were seeded in the periphery of modified 100-mm culture dishes that were made by bonding the bottoms of 60-mm culture dishes upside down into the center of 100-mm culture dishes using medical adhesive (BioGlue, Cryolife, Kennesaw, USA). Dishes were sterilized under UV light for 2 h and then coated with 1% porcine gelatin prior to plating of cells. CFDA-SE Labeled THP1 cells were then added to the “activated” HUVEC monolayer with TNFα (10 ng/mL, 16 h) prior to the addition of labeled monocytes as above described for static conditions. Adhesion was performed under orbital shear stress for 20 min at 37 °C using an orbital shaker (Infors HT Minitron incubator shaker). Shear stress was calculated using the formula: τ_max_ = *a*√ρη(2π*f*)^3^, where *a* is the radius of orbital rotation (2.5 cm), ρ is the density of the medium (1.0 g/mL), η is the viscosity of the medium (7.5 × 10^−3^ dynes·s/cm^2^) and *f *is the frequency of rotation (rotations/second). Using this formula, a shear stress of 0.5, 1 and 2 dynes/cm^2^ are achieved at a rotating frequency of 17, 26 and 42 rpm. Non-adherent THP1 cells were washed twice with PBS and attached monocytes were fixed in 4% formalin. The images of adherent monocytes were acquired with a fluorescent microscope Zeiss Axio Observer A1 (three to four different fields per sample were captured). The number of adherent monocytes was counted using the Image J software.

### Migration assay

THP1 cells (1 × 10^5^ per well) were added in the trans-well insert (TC insert 12 wells, Sarstedt, Nümbrecht, Germany) with starvation medium (RPMI 1640 with 0.5% FCS and 0.2% BSA) in the upper chamber. Different stimuli were added to the lower chamber: complete medium with 20% FCS, activated HUVECs cells with TNFα (10 ng/mL) or breast cancer cells (MDA-MB-231) conditioned medium. After 6 h, migrated THP1 cells were counted in the lower chamber. To prepare breast cancer conditioned medium, MDA-MB-231 cells were kept in culture until they reached 80% confluence, then cells were cultured in starvation medium (RPMI 1640 with 0.5% FCS, 0.2% BSA) for 48 h. Afterwards, medium was collected, filtered and used as conditioned medium. MDA-MB-231 conditioned medium was used as a cytokine-rich chemoattractant source to model complex inflammatory tumor-derived signals in the migration assay.

### Trans-EC migration assay

HUVECs were cultured until confluence on top of pre-coated trans-well inserts (TC insert 12 wells, Sarstedt, Nümbrecht, Germany) and activated with TNFα (10 ng/mL) for 16 h prior to addition of THP1 cells. 1 × 10^5^ THP1 cells per well were added in the trans-well insert in starvation medium on top of HUVECs. The lower chamber was filled with complete medium (10% FCS). HUVECs and THP1 were incubated together for 6 h at 37 °C to permit trans-EC migration. Migrated THP1 cells were counted in the lower chamber.

### Quantitative RT-PCR

mRNA expression level of genes was measured by two-step quantitative Real Time-PCR as described previously [9]. *β-ACTIN* was used as reference gene. Total RNA from THP1 cells was extracted with Trizol Reagent (TR-118, Molecular Research Center, Cincinnati, USA) following the manufacturer’s protocol. Real-time PCR reaction was performed with the GOTaq^®^ qPCR Master Mix (A6001, Promega, Madison, USA) and CFX96 Real-Time PCR Detection System (Bio-Rad, Hercules, USA). The mRNA expression level of all genes was normalized to the reference gene *β-ACTIN*. All the qRT-PCR primer sequences are shown in Table 2.

**Table 2:** RT-qPCR primer sequences

Gene	Forward primer sequences (5′−3′)	Reverse primer sequences (5′−3′)
*h-CCR2*	TGG CTG TGT TTG CTT CTG TC	TCT CAC TGC CCT ATG CCT CT
*h-IL-1β*	TCT TCG ACA CAT GGG ATA ACG A	TCC CGG AGC GTG CAG TT
*h-IL-6*	GGC ACT GGC AGA AAA CAA CC	GCA AGT CTC CTC ATT GAA TCC
*h-tnf-α*	CCC AGG GAC CTC TCT CTA ATC A	GTC ACA GGC TTG TCA CTC GG
*h-tgf-β*	CCC AGC ATC TGC AAA GCT C	GTC AAT GTA CAG CTG CCG CA
*h-TLR4*	CCC TGA GGC ATT TAG GCA GCT A	AGG TAG AGA GGT GGC TTA GGC T
*h-β-actin*	TGG CAC CCA GCA CAA TGA A	CTA AGT CAT AGT CCG CCT AGA AGC A

### ELISA

Cytokine (IL-1β, IL-6, TNFα and TGFβ) concentrations in the conditioned medium from the THP1^*WT*^ and THP^*ARG2–/–*^ cells with or without priming with LPS (100 ng/mL) for 24 h, were measured using ELISA kits (section “reagents and antibodies” for details) according to the manufacturer’s instructions. Absorbance readings minus background were converted to picograms using standard curves from the kit. Results were represented as pg/mL.

### Confocal immunofluorescence microscopy

Immunofluorescence staining was performed on mouse aortic roots. Cryosection mouse tissue was obtained from previous experiments [9] performed in the same laboratory. 10‐week‐old male *ApoE*^*–/–*^*Arg2*^+*/*+^ and *ApoE*^*–/–*^*Arg2*^*–/–*^ mice were fed either the High fat diet or a high‐cholesterol diet for 10 weeks. At 20 weeks of age, animals were anesthetized with ketamine (100 mg/kg IP) and xylazine (10 mg/kg IP), and the entire aorta from the heart to the iliac bifurcation was removed and dissected free from fat and adhering perivascular tissue. Briefly, 7‐μm cryosections of aortic roots were washed two times with PBS and fixed with 4% paraformaldehyde (pH 7.0) for 10 min at room temperature. After fixation, antigen retrieval in citrate buffer (10 mmol/L, pH 6.5) was applied in a microwave oven. Sections were rinsed 2 times with PBS, permeabilized with 0.1% Triton X-100 5–10 min at room temperature and then blocked for 1 h with PBS containing 1% BSA, 10% goat serum and 0.05% Tween-20 at room temperature. The sections were then incubated overnight at 4 °C in a dark/humidified chamber with primary antibodies, washed three times with PBS (0.05% Tween-20) and subsequently incubated for 1.5 h with the secondary antibodies diluted in blocking buffer. All sections were finally counterstained with 300 nmol/L DAPI for 5 min and autofluorescence was reduced by using Vector TrueVIEW Autofluorescence Quenching Kit. Immunofluorescent images were acquired using a Leica TCS SP5 confocal laser scanning microscope under identical acquisition settings (laser intensity, gain, and offset) for all experimental groups to allow quantitative comparison. Image analysis was performed using Image J (NIH). For single-marker analysis, mean fluorescence intensity within the region of interest (ROI) was used as the primary readout, normalised to the total aortic root area within the section to account for differences in tissue size and to approximate relative expression per cell. Colocalization analysis (CCR2, α4, αL, and αM with IBA1) was performed on images using fixed intensity threshold for each staining combination. Threshold for total signal and for the colocalised signal were determined based on the signal distribution of each marker and then applied consistently across all images acquired within the same experimental session. Colocalization was quantified as the proportion of total marker signal overlapping with the second marker (IBA1), expressed as the ratio of colocalised integrated density (IntDen)/total IntDen. Ratios were finally expressed as fold change relative to ApoE^–/–^Arg2^+/+^ mice (SK control group). This approach provided an estimation of target protein expression within IBA1-positive cells. Antibodies used are shown in Table 1.

### Statistics

Statistical analysis was performed using GraphPad Prism 10.4.1 (La Jolla, CA, USA). Data were presented as mean ± S.D. Data distribution was determined by the Kolmogorov–Smirnov test, and statistical analysis for normally distributed values was performed with analysis of variance (ANOVA) with Fisher’s LSD test. For non-normally distributed values, the Kruskal–Wallis test or Wilcoxon signed-rank test were used. Differences in mean values were considered significant at a two-tailed, and the following was used to define significant differences: * *p* ≤ 0.05, ** *p* ≤ 0.01, *** *p* ≤ 0.001, **** *p* ≤ 0.0001.

## Results

### Monocytes deficient in *ARG2* exhibit decreased integrin levels and adhesion activities onto activated endothelial cells

**Fig. 1:**
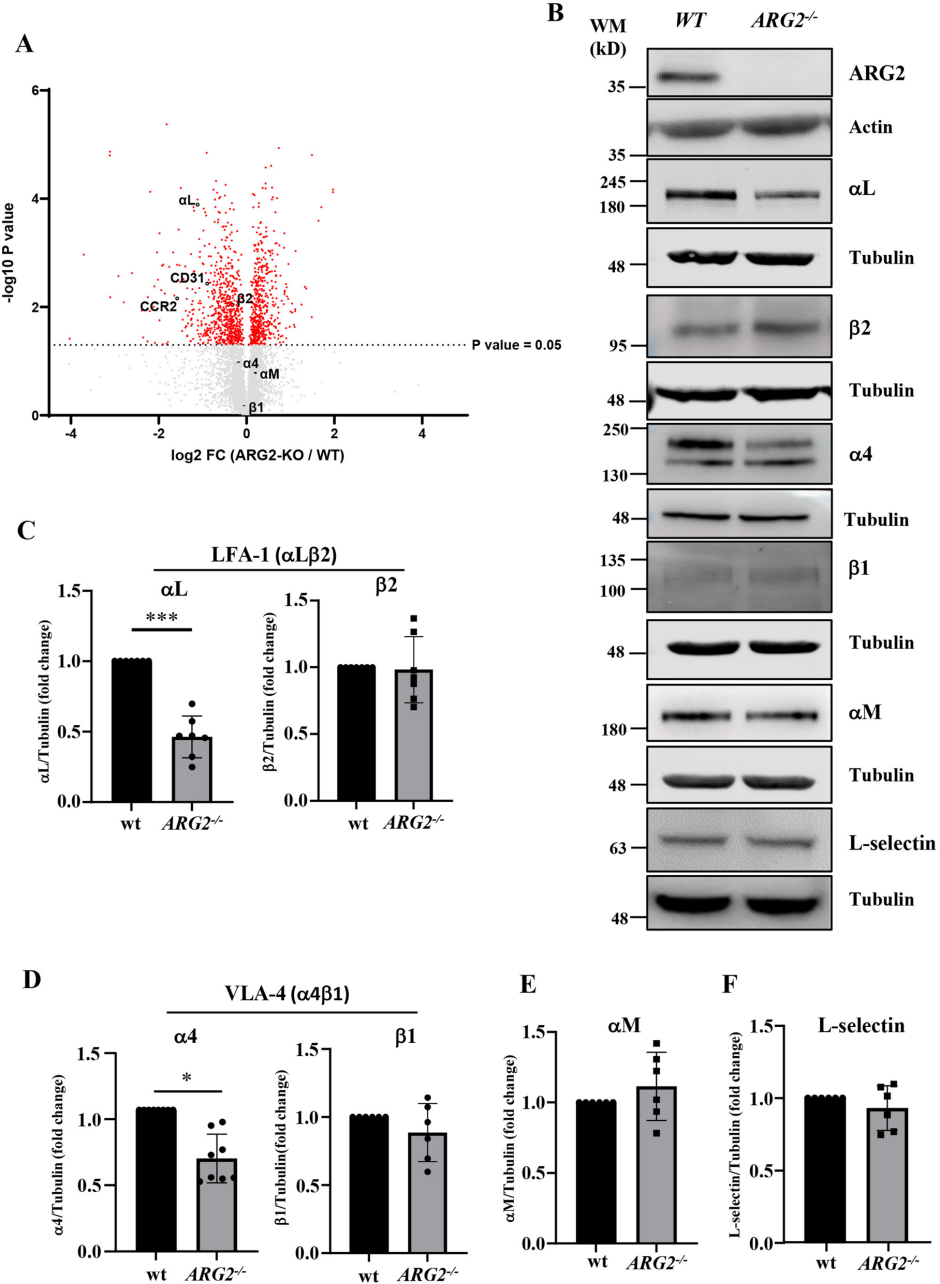
ARG2 enhances integrin levels in monocytes. **A** Volcano plot illustrating the global proteomic changes in THP1^*WT*^ versus THP1^*ARG2*–/–^ cells. Proteins surpassing significance and fold-change cutoffs are marked in red. Factors of interest affected by *ARG2*^–/–^ are indicated. Values from 0 to 4 are protein upregulated by *ARG2* ablation, and from 0 to −4 are protein reduced by *ARG2* gene knockout (n = 3). **B** Immunoblotting analysis of ARG2, the integrin α4, β1, αL, β2, αM and L-selectin in THP1^*WT*^ versus THP1^*ARG2*–/–^ cells; Tubulin served as protein loading control. Molecular weight (kD) is indicated at the left side of the blots. The plot bar graphs show the quantification of αL and β2 **C**; together forming the heterodimer LFA-1), α4 and β1 **D**; together forming the heterodimer VLA-4), αM **E**, and L-selectin **F** in each group (n = 6–8). Data are presented as mean ± SD and expressed as fold change relative to the THP1^*WT*^ group. *p ≤ 0.05, ***p ≤ 0.001 for the indicated comparisons. *WT*, wild-type; *ARG*2^–/–^, *ARG2*-knockout

Analysis of the proteomics presented as volcano plot (Fig. 1A) reveals a clear subset of proteins with significant changes in abundance between THP1^*WT*^ and THP1^*ARG2–/–*^ cells. Notably, several proteins linked to monocyte recruitment and adhesion to endothelial cells, including CCR2, PECAM1 (CD31), αL (ITGAL) and β2 (ITGB2) integrins, are downregulated (Fig. 1A). The downregulation of these factors suggests attenuation of pathways involved in chemokine responsiveness and adhesion of the THP1^*ARG2–/–*^ cells, which guided our focus toward examining this functional cluster in more detail.

**Fig. 2:**
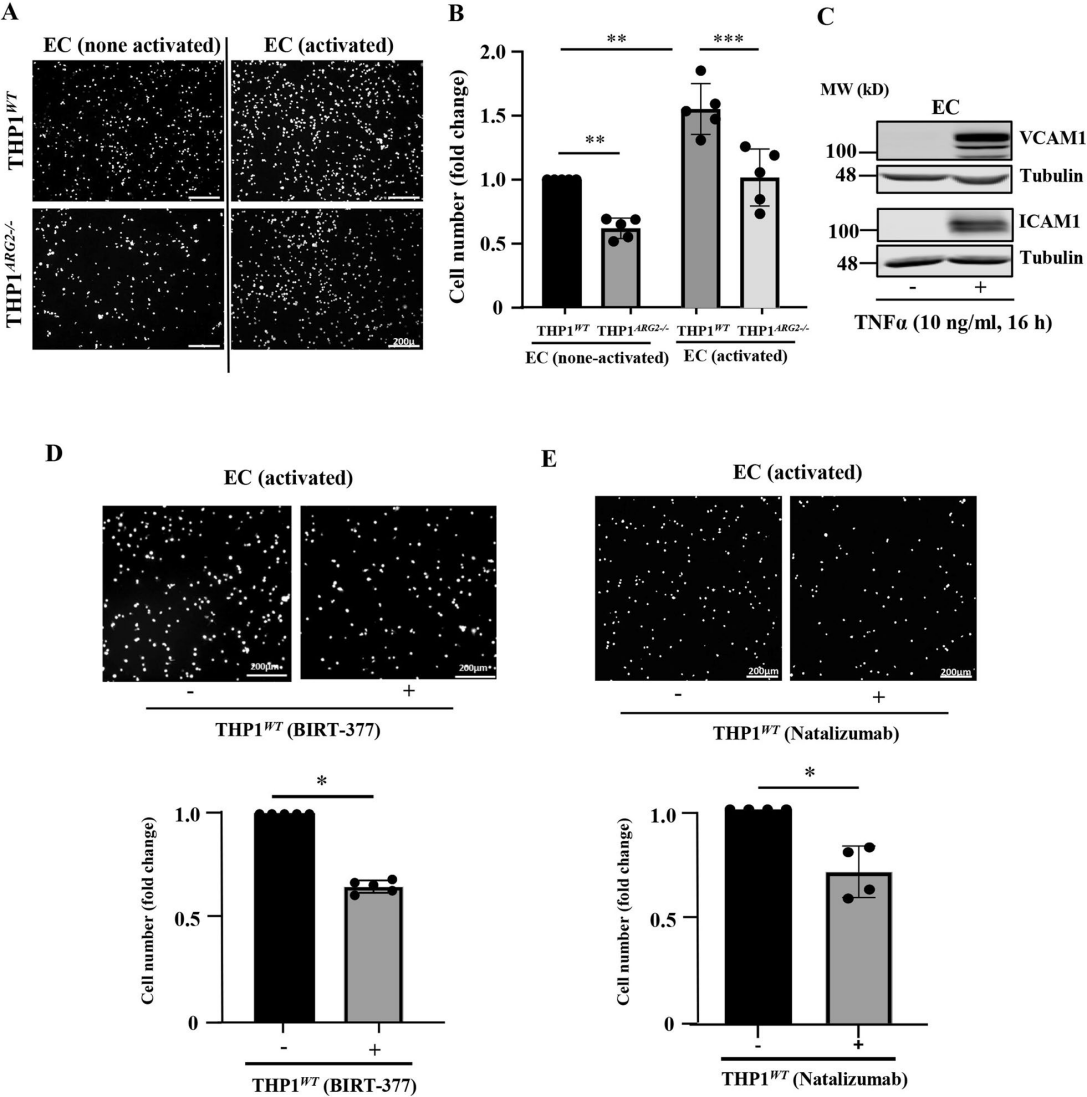
ARG2 promotes monocyte-endothelial interactions and migratory activity. **A** Representative fluorescence microscopy images showing adhesion of THP1^*WT*^ and THP1^*ARG2*–/–^ cells to non-activated and TNFα-activated HUVEC cells (EC). Scale bar = 200 µm. **B** Quantification of THP1 cell adhesion to non-activated and activated EC. **C** Immunoblot analysis of VCAM-1 and ICAM-1 levels in EC following TNFα stimulation (10 ng/mL, 16 h); Tubulin serves as loading control. Molecular weights (kD) are indicated. **D** Representative fluorescence images showing adhesion of THP1^*WT*^ cells to ECs with or without the LFA-1 blocker BIRT-377 (6 h at 20 μmol/L); **E** Representative fluorescence images showing adhesion of THP1^*WT*^ cells to ECs with or without the neutralizing antibody against α4/VLA-4 (Natalizumab) during 2 h at 15 μg/mL followed by cell adhesion assay. Corresponding quantification is shown at the bottom. Data are presented as mean ± SD and expressed as fold change relative to the THP1^*WT*^ group; n = 5. *p ≤ 0.05, **p ≤ 0.01, ***p ≤ 0.001 for the indicated comparisons. *WT*, wild-type; *ARG2*^*–/–*^, *ARG2*-knockout

Validation of the proteomic data was then performed by immunoblotting. In the THP1^*ARG2–/–*^ cells, decreased protein levels of αL and α4 integrins as compared to the THP1^*WT*^ cells are observed, while no significant changes in αM, β1, β2, and L-selectin are shown by depletion of *ARG2* in the monocytes (Fig. 1B–F). In line with these results, adhesion of THP1^*ARG2–/–*^ cells onto non-activated human endothelial cells is significantly reduced as compared to the THP1^*WT*^ cells (Fig. 2A, B). When the endothelial cells are activated by TNFα (10 ng/ml, 16 h) to express high levels of the adhesion molecules VCAM-1 and ICAM-1 (Fig. 2A, C), an increase in THP1^*wt*^ cell adhesion onto the endothelial cells is observed as expected (Fig. 2A, B), which is reduced with THP1^*ARG2–/–*^ cells (Fig. 2A, B). The importance of LFA-1 (αLβ2) for monocyte-endothelial interaction is confirmed by the results showing decreased THP1^*wt*^ adhesion onto the activated HUVECs by the LFA-1 blocker (BIRT-377) (Fig. 2D). Similarly, the neutralizing antibody against VLA-4 (Natalizumab) also reduces the monocyte adhesion to the endothelial cells (Fig. 2E), demonstrating the functional role of VLA-4 in the monocyte-endothelial interaction.

As monocyte adhesion and trans-endothelial migration in vivo occur under dynamic flow conditions, shear stress was introduced to mimic the in vivo physiological flow conditions. Under flow conditions, the adhesion of THP1^*wt*^ cells onto the TNFα–activated HUVECs as above described, is dose-dependently reduced with the increase in shear stress from 0.5 dyn/cm^2^ to 2.0 dyne/cm^2^ (Suppl. Figure  2 A to 2 C, left panels). The adhesion activities of the monocytes onto the endothelial cells under each shear stress condition are significantly reduced with the THP1^*ARG2–/–*^ cells (Suppl. Figure  2 A to 2 C, right panels). The lower adhesion activity of the THP1^*ARG2–/–*^ cells as compared to the control THP1^*wt*^ cells under the same levels of shear stress conditions re-enforces the role of ARG2 in monocyte adhesion activities to the vascular endothelial cells independently of the hemodynamic changes.

### Monocytes deficient in *ARG2* exhibit decreased migratory and trans-endothelial activities

**Fig. 3:**
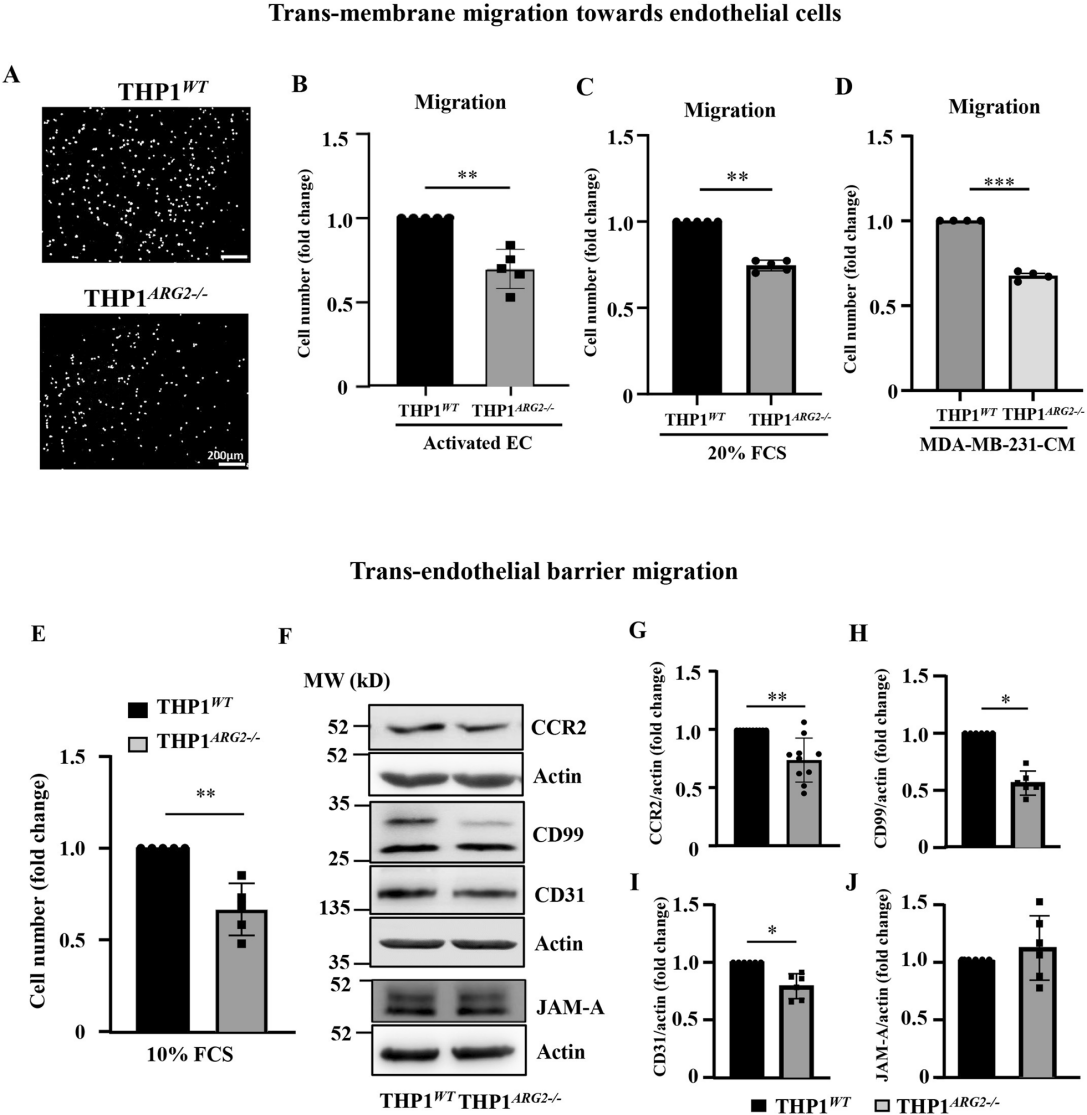
ARG2 promotes monocyte trans-endothelial activity and surface protein/receptor expression. The in vitro transwell (pore size: 6 µm) system is used to assess monocyte migratory activity, with THP1^*WT*^ or THP1^*ARG2*–/–^ placed in the upper chamber and TNFα–activated endothelial monolayer (EC) in the lower chamber. **A** Representative fluorescence images showing THP1^*WT*^ and THP1^*ARG2*–/–^ cells migrating through the transwell membrane toward activated ECs. Scale bar = 200 µm. Quantification of migrating THP1^*WT*^ and THP1^*ARG2*–/–^ cells toward **B** activated EC, **C** 20% FCS as chemoattractant in the absence of EC, and **D** breast cancer cell-conditioned medium (MDA-MB-231 CM). n = 4 or 5. Data are presented as mean ± SD and expressed as fold change relative to the THP1^*WT*^ group; The in vitro transwell system is used to assess monocyte trans-endothelial migration. THP1^*WT*^ or THP1^*ARG2–/–*^ cells were placed in the upper chamber on the membrane with 6 µm pore size containing a TNFα–activated endothelial monolayer (EC), while 10% FCS in the lower chamber served as a chemoattractant; **E** Quantification of THP1^*WT*^ and THP1^*ARG2*–/–^ cells migrating toward 10%FCS; **F** Immunoblot analysis of CCR2, CD99, CD31, and JAM-A in the THP1^*WT*^ and THP1^*ARG2*–/–^ monocytes. Actin serves as loading controls. Molecular weights (kD) are indicated. Corresponding quantifications for CCR2 **G**, CD99 **H**, CD31 **I**, and JAM-A **J** are shown; Data are presented as mean ± SD and expressed as fold change relative to the THP1^*WT*^ group. n = 4 to 10. *p ≤ 0.05, **p ≤ 0.01, ***p ≤ 0.001 for the indicated comparisons. *WT*, wild-type; *ARG2*^*–/–*^, *ARG2*-knockout

To further investigate the monocyte migratory and trans-endothelial activities, the trans-well cell culture system was used. THP1^*WT*^ or THP1^*ARG2–/–*^ cells were cultured in the upper chamber, whereas endothelial cells pre-activated with TNFα (10 ng/mL, 16 h) were cultured in the lower chamber. TNFα stimulation, which is known to stimulate endothelial cell expression of adhesion molecules (see Fig. 2C), cytokines, chemokines, and chemoattractants for leukocytes recruitment [14, 15], was used to mimic the inflamed endothelium. After six hours, significantly less THP1^*ARG2–/–*^ cells migrated towards and adhered to the endothelial cells as compared to the THP1^*wt*^ cells were observed (Fig. 3A, B). To determine whether this reduction reflects a general defect in migratory capacity, additional migration assays were performed using either 20% FCS or conditioned medium from MDA-MB-231 breast cancer cells (MDA-MB-231-CM) as chemoattractants in the lower chamber without endothelial cells. In both settings, migrated and adhered THP1^*ARG2–/–*^ cells to the endothelial cells were significantly reduced compared with the THP1^*wt*^ cells (Fig. 3C, D). Because reduced migration of THP1^*ARG2–/–*^ cells was observed not only toward TNFα-activated endothelial cells but also toward FCS and MDA-MB-231-CM, these findings indicate an intrinsic impairment of monocyte migratory capacity associated with *ARG2* deficiency.

To assess monocyte trans-endothelial activities, the THP1^*wt*^ or THP1^*ARG2–/–*^ cells were added to the TNFα-activated endothelial monolayers cultured in the upper chamber on the membrane with 6 m pores in the trans-well system, and 10% FCS was added to the lower chambers as a chemoattractant. In this assay, the endothelial cells were pre-activated by TNFα as above described to generate the inflamed endothelial barrier that supports monocyte adhesion and diapedesis. After two hours, significantly fewer THP1^*ARG2–/–*^ cells traversed the endothelial monolayer and migrated into the lower chamber compared with THP1^*wt*^ cells (Fig. 3E). The reduced trans-endothelial migratory activities of the THP1^*ARG2–/–*^ cells are associated with lower expression levels of CCR2, CD99, and CD31 (PECAM1), whereas JAM-A expression was not significantly altered as compared to THP1^*wt*^ cells (Fig. 3F–J).

### Effects of LPS on monocytic integrin expression and cytokine production

**Fig. 4:**
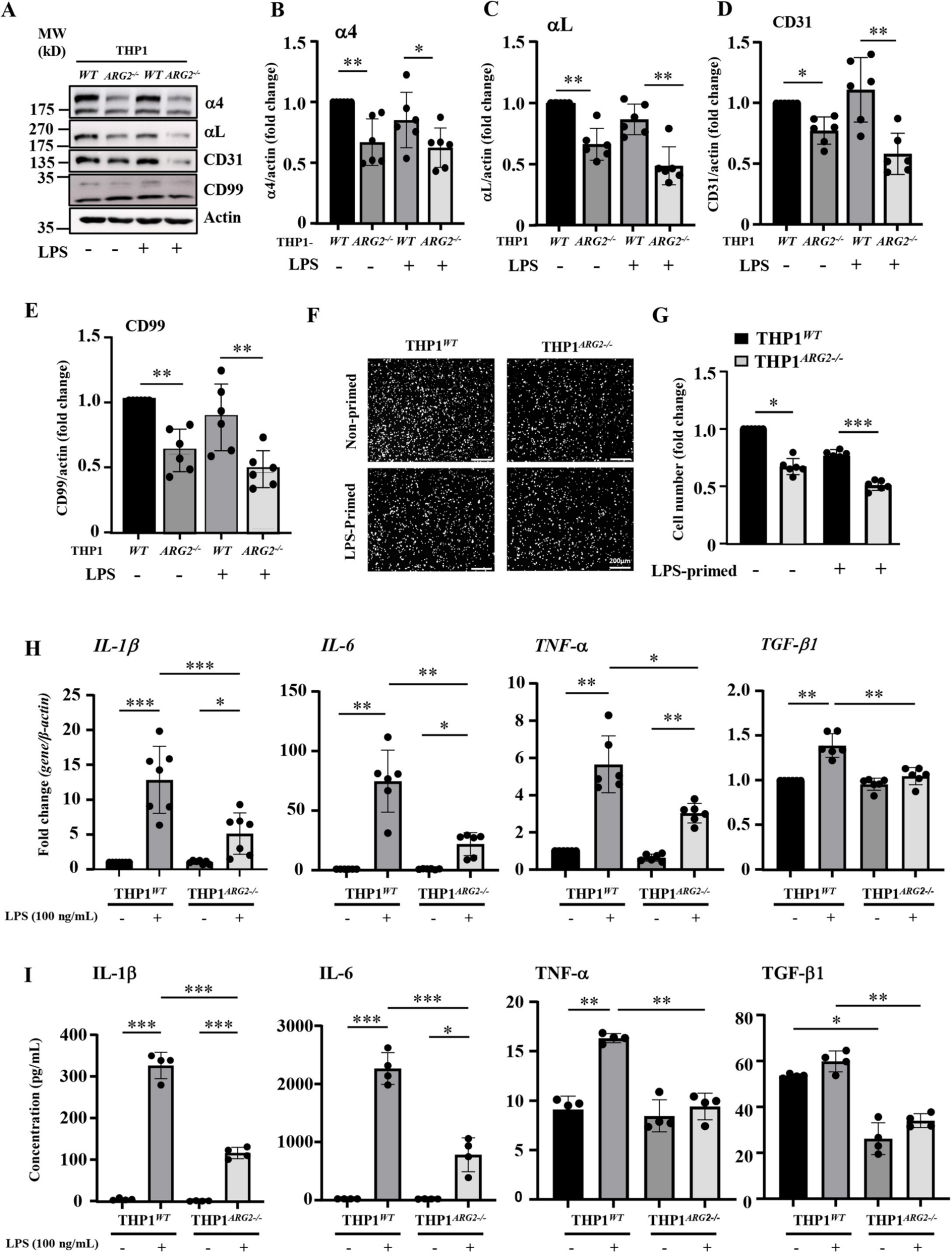
Effects of LPS on monocytic integrin and surface protein/receptor expression and cytokine production. **A** Immunoblot analysis of integrin α4, αL, CD31, and CD99 in THP1^*WT*^ and THP1^*ARG2*–/–^ cells under control conditions or following LPS stimulation (100 ng/mL, 24 h); Actin served as a loading control. Molecular weights (kD) are indicated. Quantifications of α4 **B**, αL **C**, CD31 **D**, and CD99 **E** are shown; **F** Representative fluorescence microscopy images showing adhesion of non-primed and LPS-primed THP1^*WT*^ and THP1^*ARG2*–/–^ cells to TNFα-activated endothelial cells. Scale bar = 200 µm. **G** Quantification of monocyte adhesion to the endothelial cells; n = 6 per experiment. **H** qRT-PCR analyzing mRNA expression levels of *IL-1β*, *IL-6*, *TNF-α* and *TGF-β1* in the THP1^*WT*^ and THP1^*ARG2*–/–^ monocytes under control conditions or following LPS stimulation (100 ng/mL, 24 h); *actin* served as the reference gene (n = 6 per group). mRNA levels are expressed as fold change relative to the THP1^*WT*^ control cells. Data are presented as mean ± SD; **I** ELISA analyzing IL-1β, IL-6, TNF-α, and TGF-β1 protein levels in conditioned medium from the THP1^*WT*^ and THP1^*ARG2*–/–^ monocytes under control and LPS-stimulated conditions. Data are presented as concentrations (pg/mL) of the cytokines. n = 4 per group. *p ≤ 0.05, **p ≤ 0.01, ***p ≤ 0.001 for the indicated comparisons. *WT*, wild-type; *ARG2*^*–/–*^, *ARG2*-knockout

We further investigated whether activation of the monocytes by LPS would affect their integrin and membrane receptor expression levels and whether this is regulated by *ARG2*. Stimulation of the endothelial cells with LPS (100 ng/mL, 24 h) has no significant effects on α4, αL, CD31, and CD99 (Fig. 4A–E). Independently of LPS, THP1^*ARG2–/–*^ cells reveal reduced levels of these integrins and membrane receptors (Fig. 4A–E), confirming the requirement of *ARG2* for the integrin and membrane receptors expression in the monocytes. In accordance with these results, there is no difference in adhesion of LPS-primed and non-primed THP1 cells on the TNFα-activated endothelial cells (Fig. 4F, G), while deficiency in *ARG2* decreases the cell adhesion activities under both conditions (Fig. 4F, G).

**Fig. 5:**
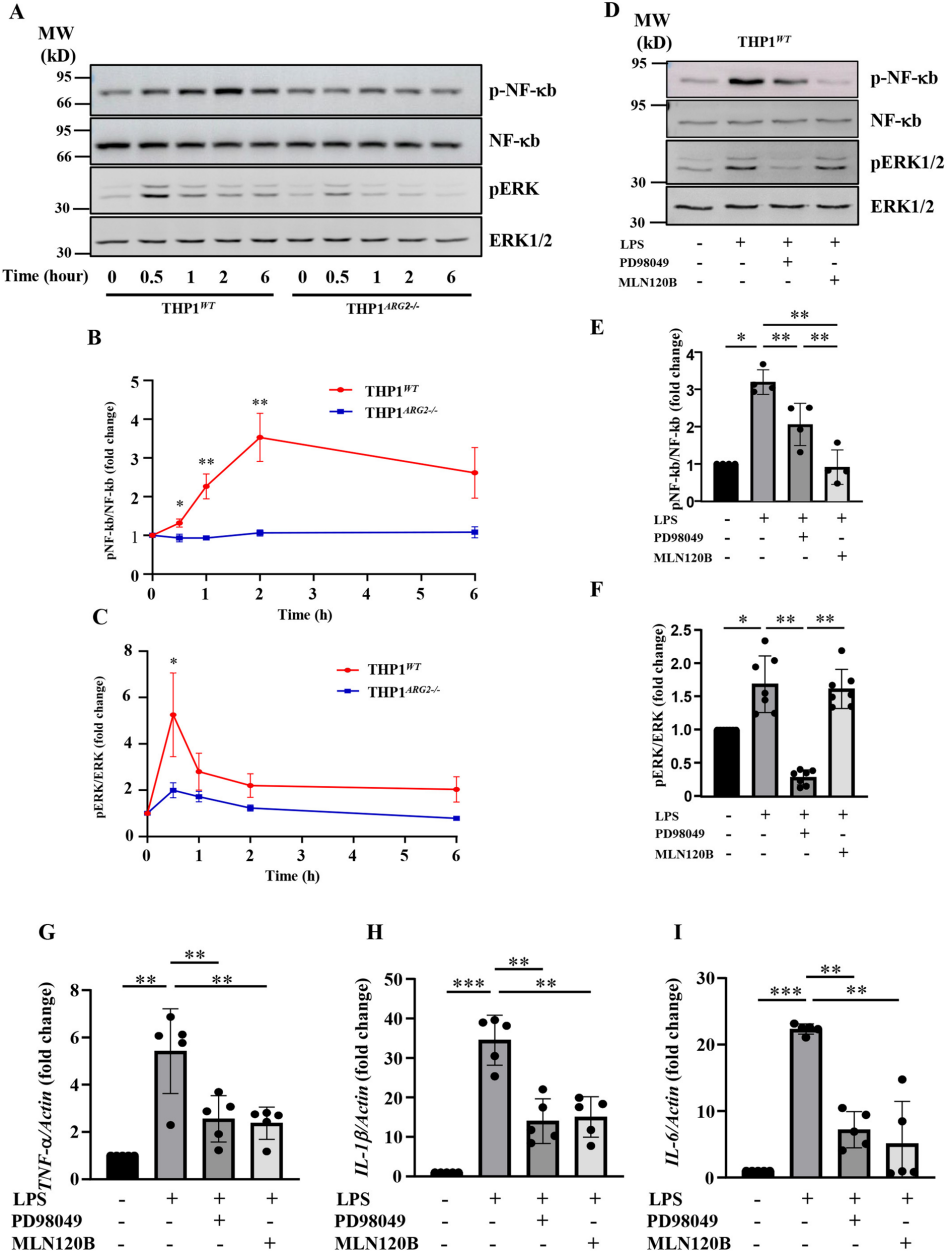
ARG2 regulates cytokine production via activation of p42/44 ERK and NF-κB signaling pathways. **A** Immunoblot analysis of total and phosphorylated NF-κB and p42/44 ERK (ERK1/2) in the THP1^*WT*^ and THP1^*ARG2*–/–^ monocytes following LPS stimulation for different times (100 ng/mL, 0 to 6 h). Molecular weights (kD) are indicated. Quantification of the time course of phosphorylated NF-κB (pNF-κB) **B** and phosphorylated ERK (pERK) **C** is shown; data are expressed as fold change of the phospho-to-total protein ratio (n = 5 per group); **D** Immunoblot analysis of total and phosphorylated NF-κB and ERK in the THP1^*WT*^ cells stimulated with LPS in the absence or presence of the NF-κB inhibitor MLN120B (20 µM, 2 h) or the ERK inhibitor PD98059 (10 µM, 30 min). Quantifications of pNF-κB **E** and pERK **F** are shown (n = 4–7 per group). qRT-PCR analyzing mRNA expression levels of *TNF-α* **G**, *IL-1β* **H**, and *IL-6* **I** in the THP1^*WT*^ monocytes under control conditions or following LPS stimulation (100 ng/mL, 24 h) in absence or presence of PD98059 (10 µM, 24 h) or MLN120B (20 µM, 24 h). n = 5 per group. Data are presented as mean ± SD and expressed as fold change relative to the THP1^*WT*^ control cells. *p ≤ 0.05, **p ≤ 0.01, ***p ≤ 0.001 for the indicated comparisons. *WT*, wild-type; *ARG2*^*–/–*^, *ARG2*-knockout

While LPS has no effect on integrin expression in both THP1^*WT*^ and THP1^*ARG2–/–*^ cells, LPS significantly enhances mRNA levels of IL-1β, IL-6, TNF-α, and TGF-β, in THP1^*WT*^ cells, which are reduced in the THP1^*ARG2–/–*^ cells (Fig. 4H). In accordance with the mRNA levels, the protein levels of these cytokines released into the conditioned medium of THP1^*WT*^ were significantly elevated by LPS, which is reduced in the conditioned medium from THP1^*ARG2–/–*^ cells (Fig. 4I). Given the central role of monocyte/macrophage inflammatory responses in vascular inflammation, we investigated whether ARG2 modulates canonical inflammatory signaling pathways, particularly ERK1/2 (p42/44 MAPK) and NF-κB, which are key downstream mediators of TLR4 activation. Following LPS stimulation over six hours, activation of both ERK1/2 and NF-κB was observed in THP1 cells, with significantly stronger and more sustained activation in THP1^*WT*^ cells compared with THP1^*ARG2–/–*^ cells (Fig. 5A–C). Peak activation occurred at approximately 2 h for NF-κB and 30 min for ERK1/2. Pharmacological inhibition experiments revealed that blockade of ERK signaling with PD98059 significantly reduced NF-κB activation, whereas inhibition of NF-κB using MLN120B had no effect on ERK phosphorylation (Fig. 5D–F), indicating that ERK acts upstream, at least in part, of NF-κB activation in LPS-stimulated THP1 cells. Consistently, inhibition of either ERK or NF-κB significantly reduced LPS-induced expression of pro-inflammatory cytokines including TNF-α, IL-1β, and IL-6 in THP1^*WT*^ cells **(**Fig. 5G–I). These results suggest that ARG2 regulates inflammatory cytokine release at least in part through modulating ERK1/2 and NF-κB signaling pathway in THP1 cells. In addition, THP1^*ARG2–/–*^ cells exhibited reduced TLR4 expression compared with THP1WT cells (Suppl. Figure 3). suggesting a role of ARG2 in TLR4 regulation in the cells.

**Fig. 6:**
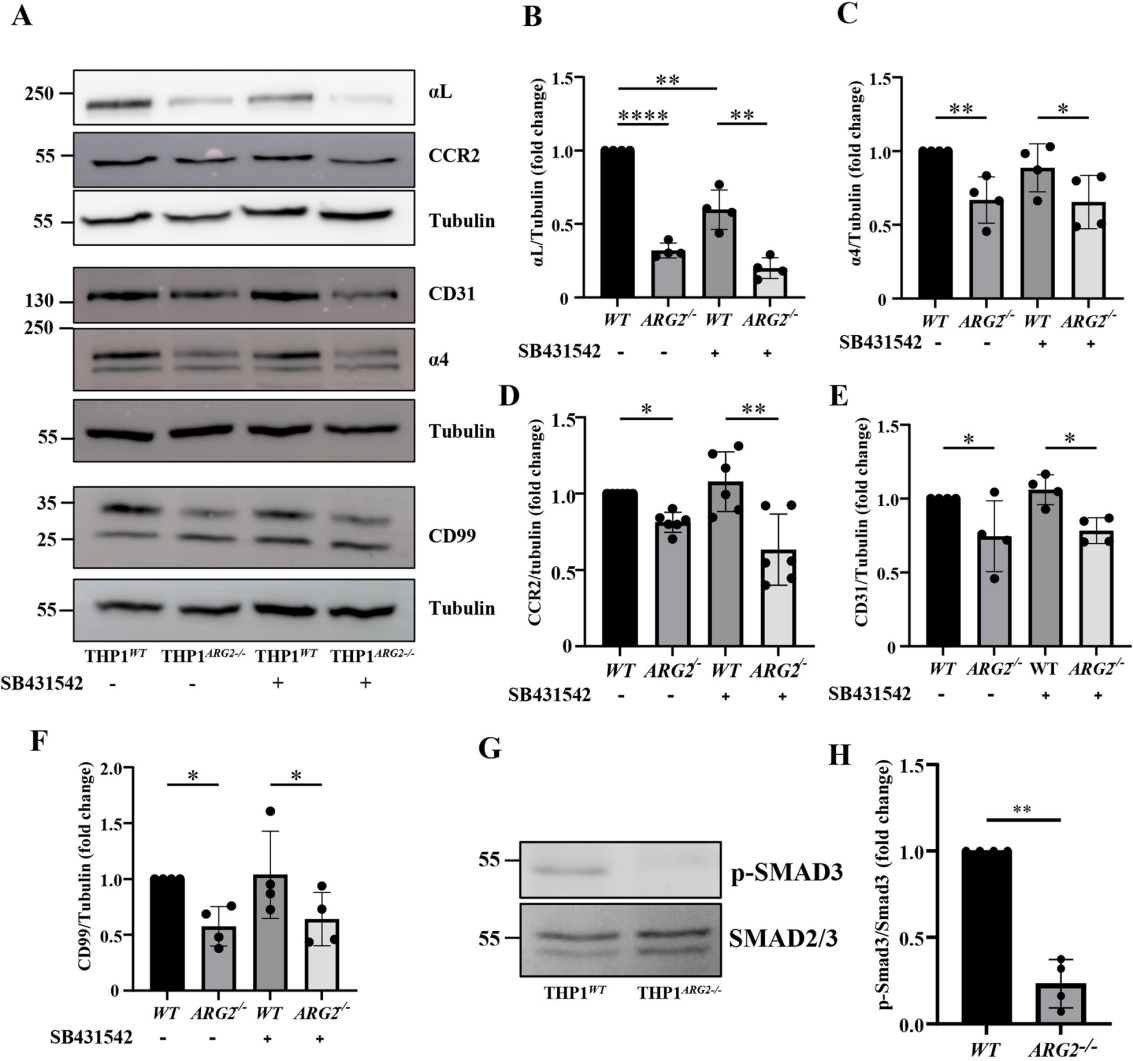
ARG2 deficiency modulates integrin αL expression via TGF‑β/SMAD signaling in THP‑1 cells. **A** Representatives immunoblot analysis of integrin subunits αL and α4, CCR2, CD31, and CD99 in THP1^*WT*^ and THP1^*ARG2*–/–^ monocytes under control conditions or following treatment with the TGF‑β receptor inhibitor SB431542 (10 μmol/L, 72 h). Tubulin was used as a loading control. Molecular weights (kDa) are indicated. Densitometric quantification of αL **B**, α4 **C**, CCR2), CD31 **E**, and CD99 **F** protein levels normalized to tubulin (n = 4–6 independent experiments); **G** Immunoblot analysis of phosphorylated SMAD3 (p‑SMAD3) and total SMAD2/3 protein levels in THP1^*WT*^ and THP1^*ARG2*–/–^ cells under baseline conditions; **H** Quantification of p‑SMAD3 levels normalized to total SMAD3 protein (n = 4 independent experiments). Data are presented as mean ± SD. *p ≤ 0.05, **p ≤ 0.01, ****p ≤ 0.0001 for the indicated comparisons. *WT*, wild-type; *ARG2*^*–/–*^, *ARG2*-knockout

We next examined whether ERK/NF-κB signaling contributes to the regulation of adhesion molecules and chemoattractant receptors altered by ARG2 deficiency. However, pharmacological inhibition of ERK or NF-κB did not significantly affect the expression of αL, α4 integrins, CD31, CCR2, or CD99 in either THP1^*WT*^ or THP1^*ARG2–/–*^ cells (Suppl. Figure 4). Since an enhanced WNT signaling pathway is also observed in the THP1^*WT*^ cells as compared to THP1^*ARG2–/–*^ cells (data not shown), the possible role of WNT signaling in the integrins and membrane receptors was also studied. Inhibition of WNT signaling pathway by IWP4 (100 nmol/L) has, however, no effects in the THP1^*WT*^ or THP1^*ARG2–/–*^ cells (Suppl. Figure 4). In contrast, inhibition of TGF-β signaling using SB431542 (10 μmol/L) selectively reduced αL expression in THP1^*WT*^ cells (Fig. 6A, B), without affecting α4 integrin, CCR2, CD31, or CD99 (Fig. 6C–F). Moreover, activation of TGFβ mediated SMAD signaling as measured by the ratio of pSMAD3/total SMAD3 is reduced in THP1^*ARG2–/–*^ cells (Fig. 6G, H). These results suggest that TGF-β signaling selectively contributes to αL regulation downstream of ARG2, whereas other integrins and receptors are regulated through mechanisms independent of both TGF-β and ERK/NF-κB signaling.

### Monocytes deficient in *ARG2* exhibit reduced paracrine effects on endothelial cells

**Fig. 7:**
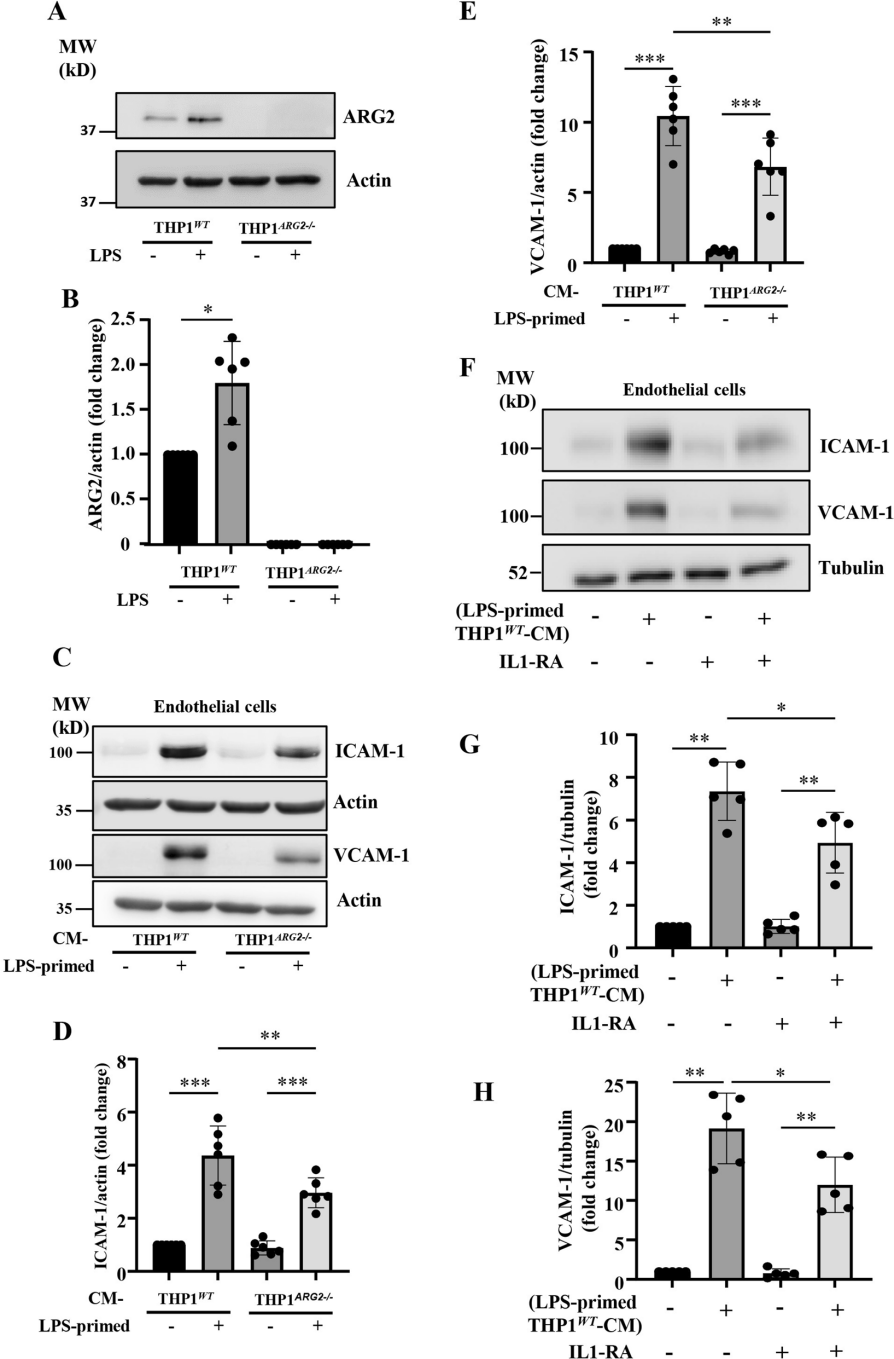
ARG2 in monocytes mediates paracrine effects on endothelial cells. **A** Immunoblot analysis of ARG2 levels in the THP1^*WT*^ and THP1^*ARG2*–/–^ monocytes following LPS stimulation (100 ng/mL, 24 h). Actin served as loading controls. Molecular weights (kD) are indicated. **B** Quantification of ARG2 protein levels; **C** Immunoblot analysis of ICAM-1 and VCAM-1 in human endothelial cells stimulated with conditioned medium derived from LPS-primed THP1^*WT*^ and THP1^*ARG2*–/–^ monocytes; Actin served as loading controls. Molecular weights (kD) are indicated. Corresponding quantifications of ICAM-1 **D** and VCAM-1 **E** are shown; **F** Immunoblot analysis of ICAM-1 and VCAM-1 in the endothelial cells stimulated with conditioned medium from LPS-primed THP1^*WT*^ cells with or without IL-1β receptor antagonist (IL-1RA) treatment. Quantifications of ICAM-1 **G** and VCAM-1 **H** are shown. n = 5–6 per group. Data are presented as mean ± SD and expressed as fold change relative to the THP1^*WT*^ cells. *p ≤ 0.05, **p ≤ 0.01, ***p ≤ 0.001 for the indicated comparisons. *WT*, wild-type; *ARG2*^*–/–*^, *ARG2*-knockout

In line with the above results, ARG2 levels in the THP1^*WT*^ cells are significantly enhanced upon stimulation with LPS (100 ng/ml, 24 h) (Fig. 7A, B). Importantly, stimulation of the endothelial cells with the conditioned medium derived from LPS-primed THP1^*WT*^ cells reveals strong upregulation of endothelial ICAM-1 and VCAM-1 (Fig. 7C–E). These effects are much weaker with the conditioned medium derived from the LPS-primed THP1^*ARG2–/–*^ cells (Fig. 7C–E), suggesting a role of ARG2 in paracrine release of factors from monocytes. Indeed, upregulation of ICAM-1 and VCAM-1 in endothelial cells by the conditioned medium from LPS-primed THP1^*WT*^ cells is significantly reduced when the endothelial cells are pretreated with the IL-1 receptor antagonist (IL1-RA) (Fig. 7F–H).

### *Arg2* deficiency reduces macrophage integrins in atherosclerosis

**Fig. 8:**
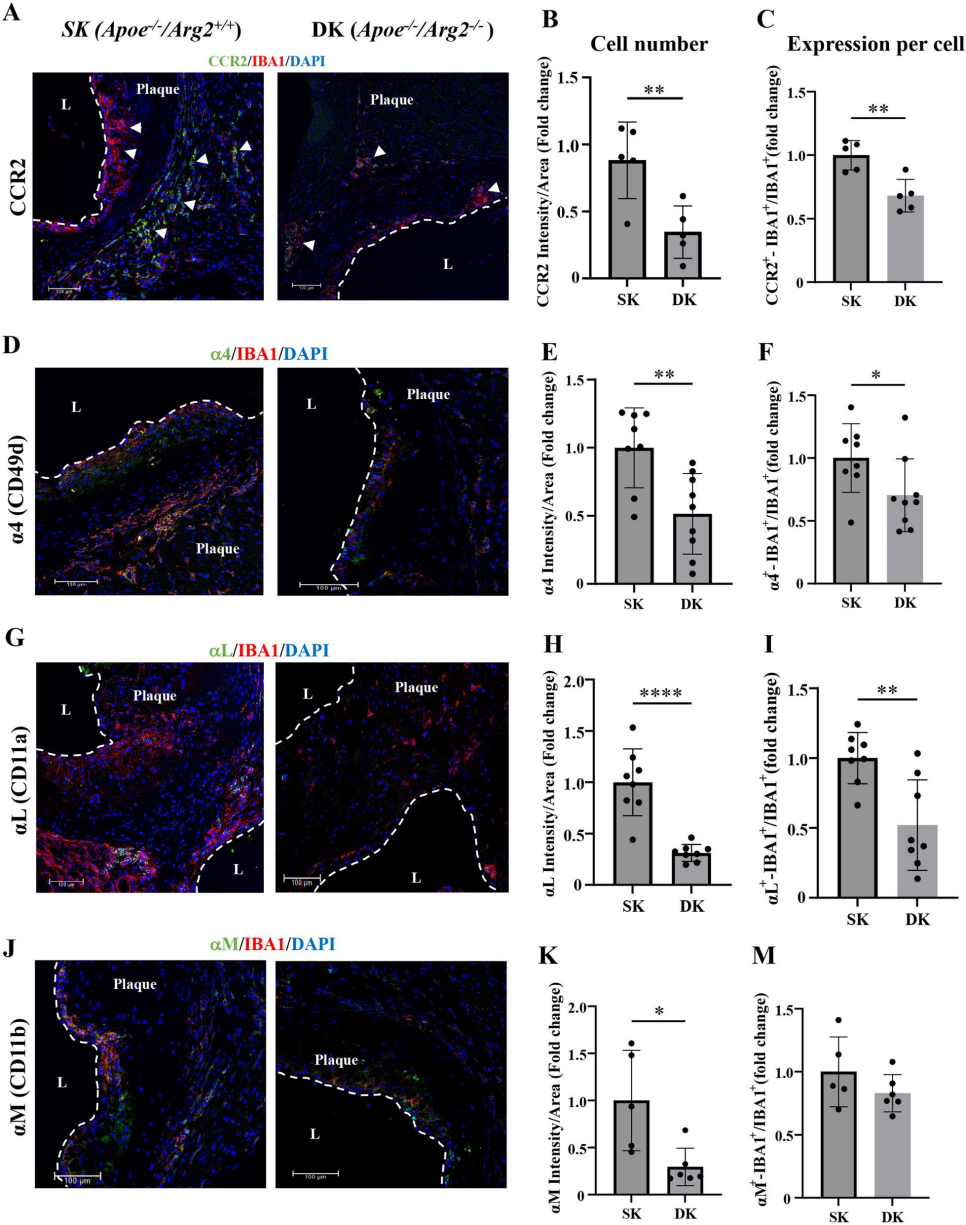
*Arg2* enhances macrophage CCR2 and integrin expression in atherosclerotic plaques. Representative confocal microscopic images illustrating a part of the atherosclerotic plaques from aortic roots of each genotype are presented **A, D, G and J** for the specific staining in macrophages. **A** Confocal microscopy images showing immunofluorescence double staining of CCR2 (green) and IBA1 (red; macrophage marker) of *ApoE*^*–/–*^*/Arg*^+*/*+^ (single knockout; SK) and *ApoE*^*–/–*^*/Arg*^*–/–*^ (double knockout; DK). DAPI (blue) labels nuclei. Scale bar = 100 µm. Quantification of CCR2 signal intensity per area is shown in **B**, and colocalization between CCR2 and IBA1 (reflecting CCR2 expression per macrophage and expressed as the ratio of double-positive area to total IBA1 area) is shown in **C**, **D** Confocal microscopy images showing immunofluorescence double staining of integrin α4 (green) and IBA1 (red). DAPI (blue) labels nuclei. Scale bar = 100 µm. Quantification of α4 signal intensity **E** and colocalization between α4 and IBA1 **F** is shown; **G** Confocal microscopy images showing immunofluorescence double staining of integrin αL (green) and IBA1 (red). DAPI (blue) labels nuclei. Scale bar = 100 µm. Quantification of αL signal intensity **H** and colocalization between αL and IBA1 **I** is shown; **J** Confocal microscopy images showing immunofluorescence double staining of integrin αM (green) and IBA1 (red). DAPI (blue) labels nuclei. Scale bar = 100 µm. Quantification of αM signal intensity **K** and colocalization between αM and IBA1 **M** is shown. L means vascular lumen. plaque boundaries to lumens of the aortic roots are indicated by dotted lines. Arrowheads indicate typical examples of macrophage clusters in the plaque. Each experiment was repeated with 5 to 8 animals. Data is presented as mean ± SD and expressed as fold change. *p ≤ 0.05, **p ≤ 0.01, ****p ≤ 0.0001 for the indicated comparisons

Our previous study demonstrated that *Arg2* gene knockout in the atherosclerotic *ApoE*^*–/–*^ mice, i.e., *ApoE*^*–/–*^*Arg2*^*–/–*^ double knockout mice fed high cholesterol diet reduced atherosclerotic lesion size and decreased accumulation of pro-inflammatory macrophages in the atherosclerotic plaque [9]. In the present study, we further analysed the effects of *Arg2* deficiency on relevant macrophage integrins and membrane receptors in the atherosclerotic plaques of the animal models. Immunofluorescence confocal microscopy reveals a significantly lower number of CCR2-positive cells, a hallmark of infiltrating monocytes/macrophages within the aortic roots of *ApoE*^*–/–*^*Arg2*^*–/–*^ mice compared with the *ApoE*^*–/–*^*Arg2*^+*/*+^ single knockout mice (Fig. 8A, B). Co-localization analysis of CCR2 with IBA1 (macrophage marker) further demonstrates that CCR2 expression per macrophage is reduced in the *ApoE*^*–/–*^*Arg2*^*–/–*^ mice (Fig. 8C). It is of note that CCR2^+^ macrophages are mainly localized in the less advanced plaque region, while IBA1^+^ macrophages in the advanced plaque region are CCR2 negative (Fig. 8A). In line with the observation with THP1 cells in vitro, the overall expression of macrophage-associated integrins, including α4 (Fig. 8D–F), αL (Fig. 8G–I), and αM (Fig. 8J–L), is also decreased in the plaque lesion of the *ApoE*^*–/–*^*Arg2*^*–/–*^ mice. Notably, quantification of integrin colocalization with IBA1 reveals that the *ApoE*^*–/–*^*Arg2*^*–/–*^ mice exhibit reduced α4 (Fig. 8F), αL (Fig. 8I), but not αM (Fig. 8L) in the macrophages of the atherosclerotic plaque as compared to the *ApoE*^*–/–*^*Arg2*^+*/*+^ controls, which is in agreement with the data obtained in the human THP1 cells.

## Discussion

Our previous studies showed that ARG2 in the monocytes/macrophages promotes pro-inflammatory responses and the cell infiltration in various tissues/organs under disease conditions such as atherosclerosis, obesity, and in aging [9, 16–18]. In the present study, we further explored the underlying mechanisms by showing that ARG2 regulates integrin expression in monocytes, therefore affecting the monocyte-endothelial interaction and trans-endothelial migration, which is crucial for atherogenesis.

Studies in the literature show that integrins VLA-4 (known as α4β1 integrin) and LFA-1 (known as αLβ2 integrin) on the surface of monocytes binds to VCAM-1 and ICAM-1 on the endothelial cells, particularly the inflamed endothelial cells, respectively, that ensures the firm adhesion of monocytes on the endothelial cells [2]. This process is then followed by monocyte polarization and migration to the interendothelial junctions, where LFA-1 interacts with the endothelial JAM-A, VLA-4 integrin binds to JAM-B, and MAC1 (known αMβ2-integrin) to JAM-C [2]. This cell–cell interaction allows the monocyte transmigration through tight junctions between the endothelial cells. Lastly, the hemophilic engagement of PECAM1 (CD31) and CD99 between monocytes and endothelial cells leads to monocyte extravasation [5]. In this context, we demonstrate in the current study that ablation of *ARG2* gene in the human monocytes reduces α4, αL, CD99 and CD31) levels and therefore results in the decreased monocyte adhesion, trans-endothelial migration, and extravasation as demonstrated in the in vitro cellular models. It is to note that other integrins such as L-selectin, β1, β2, αM are not affected by *ARG2* deficiency, demonstrating specific dependence of the integrins on *ARG2*.

Since ARG2 promotes monocyte/macrophage pro-inflammatory responses and stimulation of monocytes/macrophages with LPS enhances ARG2 levels in the THP1 cells as shown by our previous study [9], we investigated whether LPS could regulate the integrins. Surprisingly, stimulation of the THP1 cells with LPS does not affect the integrins levels of α4, αL, PECAM1 (CD31), and CD99 and ablation of *ARG2* reduces the integrin levels independently of LPS stimulation. These results demonstrate that the integrins of interest are not regulated by LPS-induced inflammation in the monocytes. The results also suggest that ARG2 elevation is not sufficient to enhance the integrin expression but required to maintain significant levels of the integrins to ensure monocyte-endothelial interaction during the inflammation process. In accordance with these results, the monocytes with or without LPS-priming reveal comparable adhesion activities onto endothelial cells, which are reduced by *ARG2* deficiency under both the priming and non-priming conditions.

Importantly, stimulation of monocytes with LPS markedly enhances both the mRNA and protein levels of the pro-inflammatory cytokines such as IL-1β, IL-6, TNF-α, but not TGF-β1 at the protein level, and these responses are significantly attenuated by ARG2 ablation. This observation confirms our previous results showing a pro-inflammatory role of *ARG2* in macrophages in cardiovascular disease and obesity [9]. The inflammatory cytokines are mediated by the cell membrane receptor TLR4 which senses PAMPs or DAMPs to upregulate the transcription of genes of the inflammatory cytokine and chemokine, orchestrating inflammatory responses in pathological conditions [19]. The reduced cytokine production in LPS-stimulated monocytes with *ARG2* deficiency may be associated with the decreased TLR4 expression, because *TLR4* expression is significantly lower in the *ARG2* deficient monocytes. Although the molecular mechanism linking ARG2 deficiency to reduced TLR4 expression remains to be determined, our findings suggest that ARG2 contributes to maintaining TLR4-dependent inflammatory signaling in monocytes.

Since LPS stimulates monocyte/macrophage inflammatory responses via TLRs linking to activation ERK/NFκB pathways [20, 21], the decreased TLR4 expression in the ARG2-deficient monocytes as compared to the THP1WT cells is consistent with the reduced activation of ERK/NFκB pathway and decreased expression/production of a series of pro-inflammatory cytokines in response to LPS. Interestingly, pharmacological inhibition of ERK reduces NFκB activation, while inhibition of NFκB had no effect on ERK in the monocytes/macrophages in response to LPS, demonstrating that ERKs are upstream of NFκB pathway, mediating the pro-inflammatory responses of the cells. This conclusion is further supported by the fact that pharmacological inhibition of either ERKs or NFκB pathway is able to reduce LPS-mediated upregulation of TNF-α, IL-1β, and IL-6 gene expression.

Since *ARG2* deficiency reduces ERK-NFkB signaling pathway in monocytes/macrophages as demonstrated by our current study and by other groups [22], we hypothesized that the ERK-NFkB signaling pathway in monocytes could regulate integrin expression. However, inhibition of ERK and/or NFkB does not affect the integrin protein levels. These results exclude the involvement of reduced ERK-NFkB signaling in the downregulation of integrins and membrane receptors in the ARG2-deficient THP1 cells. Importantly, these findings also indicate that integrin regulation is uncoupled from LPS-induced inflammatory signaling, since ERK/NF-κB primarily control cytokine production but not adhesion receptor expression in this system.

It has been shown that TGFβ1 enhances monocyte/macrophage integrin expression [23, 24] and TGFβ1 release is reduced by *ARG2* deficiency in the monocytes, we investigated whether ARG2 regulates integrins and membrane receptors through TGFβ1. Indeed, treatment of the *WT* cells but not ARG2^–/–^ cells with TGFβ receptor blocker reduces αL levels (not other integrins or membrane receptors). These results demonstrate that ARG2 maintains αL levels via TGFβ. In contrast, other integrins and membrane receptors (α4, CD31, CD99, CCR2) are not affected by ERK/NFκB, WNT, or TGFβ signaling inhibition, suggesting that their regulation involves additional ARG2-dependent but currently undefined mechanisms.

It remains to be investigated how *ARG2* deficiency reduces other integrin and membrane receptor levels in the monocytes. Although the reduced ERK-NFkB is not involved in the downregulation of the integrins and membrane surface receptors in the *ARG2*-deficient THP1 cells, it is responsible for reduced pro-inflammatory cytokine production as above discussed. The secreted cytokines from monocytes/macrophages can activate vascular endothelial cells to express elevated levels of adhesion molecules such as ICAM-1 and VCAM-1, which further facilitates the monocyte-endothelial interaction [25].

Indeed, conditioned-media from THP1^*WT*^ cells exert stronger upregulating effects on endothelial ICAM-1 and VCAM-1 as compared to the conditioned media from the THP1^*ARG–/–*^, demonstrating a paracrine effect of the monocytes/macrophages on endothelial inflammation. Further experiments identify that IL-1β released from activated monocytes/macrophages is, at least, partially responsible for the upregulation of endothelial adhesion molecules, because IL1-ra, the IL-1 receptor antagonist reduces this effect. However, the incomplete inhibition by IL-1ra indicates that additional ARG2-dependent soluble factors, such as TNFα or IL-6, are also likely involved in endothelial activation. This result is in line with our previous findings showing decreased atherosclerosis burden and several pro-inflammatory cytokine expression levels including IL1β in the *Apoe*^*–/–*^*Arg2*^*–/–*^ mouse model as compared to the *Apoe*^*–/–*^ mouse [9]. The role of IL-1β in atherosclerosis is also confirmed by the CANTOS trial which demonstrates the efficacy of an anti-IL-1β monoclonal antibody in patients with coronary artery diseases [26]. Together, these findings suggest that IL-1β contributes to, but does not fully account for, the enhanced endothelial VCAM-1 and ICAM-1 expression induced by ARG2-expressing monocytes. It remains to be investigated whether other cytokines released from monocytes/macrophages such as TNFα and/or IL-6 are also contributing to the enhanced endothelial VCAM-1 and ICAM-1 upregulation.

Monocytes/macrophages-EC interaction plays an essential role in acute and chronic inflammation-associated diseases such as atherosclerosis [25]. Multiple studies suggest a role for α4β1 integrins in atherosclerotic plaque formation [11, 27, 28]. Studies demonstrate that hypercholesterolemia enhances αL expression in human patients and blocking antibodies to αLβ2 significantly reduce early macrophage recruitment in the hypercholesterolemic rats [29, 30]. Moreover, atherosclerotic plaques show enhanced integrin α4β7 in macrophages [31], and α4β1 blocking antibodies significantly reduce myeloid cell recruitment and neointimal growth in atherosclerosis-prone *ApoE* knockout mice [11, 12]. In line with these studies, we demonstrate that in the atherosclerotic plaques, *ApoE*^*–/–*^*Arg2*^*–/–*^ mice on a high-cholesterol diet reveal reduced accumulation of pro-inflammatory macrophages within the plaques and also decreased expression levels of α4, αL, CCR2 in the macrophages within the plaque. This observation is consistent with our in vitro findings with the human THP1 cells and provides a mechanistic explanation for the previous finding that Arg2^*–/–*^ mice on the ApoE^*–/–*^ background reveal reduced atherosclerosiswell explain the previous finding that *Arg2*^*–/–*^ mice on the *ApoE*^*–/–*^ background reveal reduced atherosclerosis [9]. It is of note that CCR2^**−**^ macrophages are more abundant in the advanced atherosclerotic plaque region. This seems counterintuitive suggesting a decreased infiltration of monocytes into blood vessel wall, because CCR2^+^ macrophages represent infiltrated cells [32, 33]. However, taking into account that macrophages which are infiltrated into the blood vessels change their phenotype, the accumulation of CCR2^−^ macrophages could be explained by the studies showing that CCR^+^ monocytes/macrophages become CCR^−^ under differentiation and stimulation with atherogenic stimuli in atherosclerosis, once they have entered into the vascular wall [34, 35]. Furthermore, decreased levels of α4, αL, and αM were found in the atherosclerotic plaques of the *ApoE*^*–/–*^*Arg2*^*–/–*^ mice as compared to the *ApoE*^*–/–*^*Arg2*^+*/*+^ control animals, which is not only due to the lower numbers of macrophages in the plaques, but also due to the decreased levels of the integrins (except αM) in the macrophages, much alike as observed in the human THP1 monocytes. Of note, the present study utilized global *ApoE*^*–/–*^*Arg2*^*–/–*^ mice, which does not allow us to distinguish the relative contribution of monocyte/macrophage-intrinsic ARG2 from potential effects of ARG2 in other cell types, including vascular or metabolic tissues. Therefore, although our in vitro data using THP1 cells strongly support a monocyte-intrinsic role of ARG2 in regulating integrin expression, chemoattractant receptor levels, and inflammatory signaling, we cannot exclude additional contributions from non-myeloid compartments in vivo. Future studies using myeloid-specific Arg2 deletion models will be required to definitively determine the cell type-specific role of ARG2 in monocyte recruitment and atherogenesis.

## Conclusions

Our study demonstrates that the mitochondrial enzyme ARG2 is essential for expression of various integrins i.e., αL and α4, as well as membrane receptors such as CD99, CD31 and CCR2 in monocytes, involving TGFβ1-dependent regulation (for αL) and other unknown mechanisms (for α4 and the remaining receptors) contributing to atherogenesis. Moreover, ARG2 is required for TLR4-ERK-NFκB signaling and release of pro-inflammatory cytokines, which contribute to endothelial activation and further enhance monocyte adhesion and trans-endothelial migration. Importantly, the primary effect of ARG2 on monocyte-endothelial interaction is mediated through its regulation of integrin and membrane receptor expression, while cytokine-mediated endothelial activation represents an additional amplifying mechanism.

**Fig. 9:**
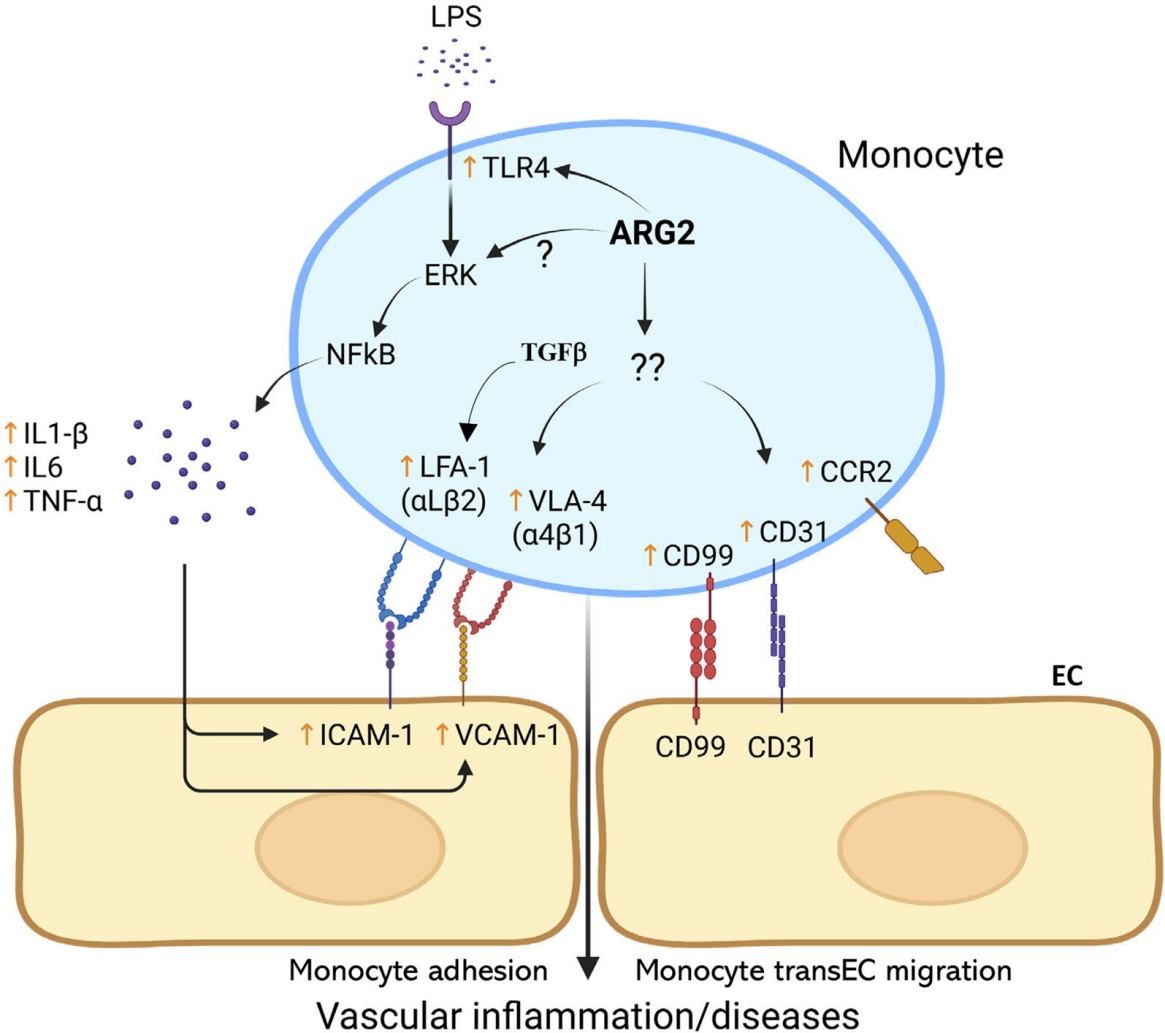
Schematic illustration of the role of ARG2 in regulation of monocytic integrins and surface proteins/receptors and interaction with endothelial cells, leading to vascular inflammation and atherosclerosis

These combined effects of ARG2 facilitate monocyte adhesion and trans-endothelial migration, contributing to atherogenesis (Fig. 9). This study has deepened our understanding of the mechanisms of monocyte-mediated vascular inflammation and disease development and identifies ARG2 as a key regulator of integrin and membrane receptor expression in monocytes. Accordingly, ARG2 may represent a promising target for future therapeutic intervention in vascular and other chronic inflammatory diseases, although studies using pharmacological ARG2 inhibition will be required to establish its therapeutic potential.

## Supplementary Information

Supplementary Material 1

Supplementary Material 2

Supplementary Material 3

Supplementary Material 4

Supplementary Material 5

## Data Availability

New dataset (proteomics) is generated and will be deposited in a repository with hyperlinks and persistent identifiers (e.g. DOI or accession number) after our manuscript is accepted/published.
