# Supplementary material for: Arginase-2 enhances monocyte-endothelial interaction through regulation of integrin and membrane receptor expression: role in atherogenesis
Source: J Biomed Sci. 2026 Jul 13;33:73. doi: 10.1186/s12929-026-01278-3 (PMC13366879; doi:10.1186/s12929-026-01278-3)
Supplement: Supplementary file 1 — Supplementary Material 1 [file 12929_2026_1278_MOESM1_ESM.pdf]

June 26, 2026
Research Article
J Biomed Sci
Submission ID: 389c2208-1185-45f2-aad2-364c62bf1d23 (R1)

Arginase-2 enhances monocyte-endothelial interaction through regulation of integrin and membrane receptor expression: Role in atherogenesis

Guillaume Ajalbert[1], Yuejun Yao[1], Sophie Rohrer[1], Matteo Caretti[1], Santhoshkumar Sundaramoorthy[1], Anastasia Rigkou[1], Michael Stumpe[2], Xiu-Fen Ming[1], Duilio M. Potenza[1]*, Zhihong Yang[1]*

[1]Laboratory of Cardiovascular and Aging Research, Department of Endocrinology, Metabolism, and Cardiovascular System, Faculty of Science and Medicine, University of Fribourg, Chemin du Musee 5, 1700 Fribourg, Switzerland; [2]Department of Biology, University of Fribourg, Chemin du Musee 10, CH-1700 Fribourg, Switzerland

*Corresponding authors

Prof. Zhihong Yang, MD.
Laboratory of Cardiovascular and Aging Research, Department of EMC, Faculty of Science and Medicine, University of Fribourg, Chemin du musée 5, 1700 Fribourg, Switzerland

Phone: +41 26 300 8593, email: zhihong.yang@unifr.ch

or

Dr. Duilio M. Potenza, PhD
Laboratory of Cardiovascular and Aging Research, Department of EMC, Faculty of Science and Medicine, University of Fribourg, Chemin du musée 5, 1700 Fribourg, Switzerland

Phone: +41 26 300 8585, Email: duilio.potenza@unifr.ch

Funding: This work was supported by the Swiss National Science Foundation (31003A_179261 and 310030_219438 to ZY).

Supplementary Figure legends

Suppl. Fig. 1: *ARG2* deficiency in THP1 cells has no effect on cell proliferation and cell viability. (**A**) Analysis of cell viability by Trypan blue staining exclusion (**B**) Analysis of cell proliferation by the MTT assay (**C**) Analysis of cell proliferation by cell number counting. n=6. *WT*, wild-type; *ARG2*$^{-/-}$, *ARG2*-knockout.

Suppl. Fig. 2: *ARG2* deficiency in THP1 cells decreases cell adhesion to endothelial cells under flow conditions. Representative fluorescence microscopy images and corresponding quantification (right) showing adhesion of the THP1^{WT} and THP1$^{ARG2-/-}$ monocytes to TNF-α-activated endothelial cells under increasing flow (shear stress) conditions: 0.5 dyns/cm² (**A**), 1 dyns/cm² (**B**), and 2 dyns/cm² (**C**). Scale bar = 200 µm. n = 5 per group. Data are presented as mean ± SD and expressed as fold change relative to the THP1^{WT} control cells. $^{**}p \leq 0.01$ for the indicated comparisons. *WT*, wild-type; *ARG2*$^{-/-}$, *ARG2*-knockout.

Suppl. Fig. 3: *ARG2* deficiency in THP1 cells decreases *TLR4* expression. qRT-PCR analysis of mRNA expression levels of *TLR4* in the THP1^{WT} and THP1$^{ARG2-/-}$ monocytes. n=6. $^{*}p \leq 0.05$. *WT*, wild-type; *ARG2*$^{-/-}$, *ARG2*-knockout.

Suppl. Fig. 4: Effects of inhibitors of ERK, NFκB, and WNT signaling on integrin expression levels. (A) Representative immunoblotting of integrin protein levels in the THP1^{WT} and THP1$^{ARG2-/-}$ cells. Quantification of αL (**B**), α4 (**C**), and CD31 (**D**) are shown. n = 3. Data are presented as mean ± SD and expressed as fold change relative to the control THP1^{WT} cells. IWP4 (100 nmol/L, 24 hours): WNT signaling inhibitor; PD98049 (10 µmol/L, 24 hours): ERK inhibitor, and MLN120B (20 µmol/L, 24 hours), NFκB inhibitor. $^{*}p \leq 0.05$, $^{**}p \leq 0.01$ for the indicated comparisons. *WT*, wild-type; *ARG2*$^{-/-}$, *ARG2*-knockout.
