# Supplementary figures and images for: Arginase-2 enhances monocyte-endothelial interaction through regulation of integrin and membrane receptor expression: role in atherogenesis
Source: J Biomed Sci. 2026 Jul 13;33:73. doi: 10.1186/s12929-026-01278-3 (PMC13366879; doi:10.1186/s12929-026-01278-3)

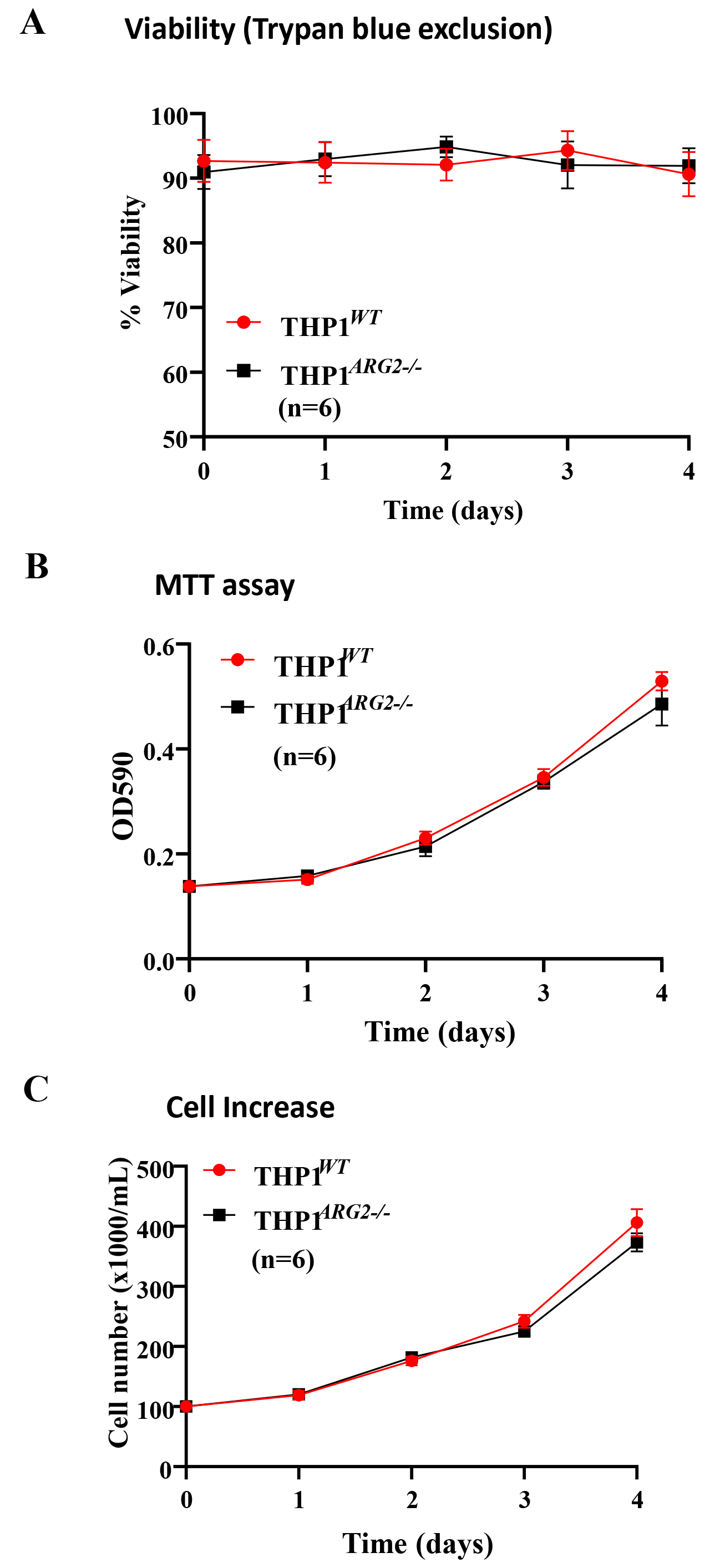

Supplement: Supplementary file 2 — Supplementary Material 2 [file 12929_2026_1278_MOESM2_ESM.tif]

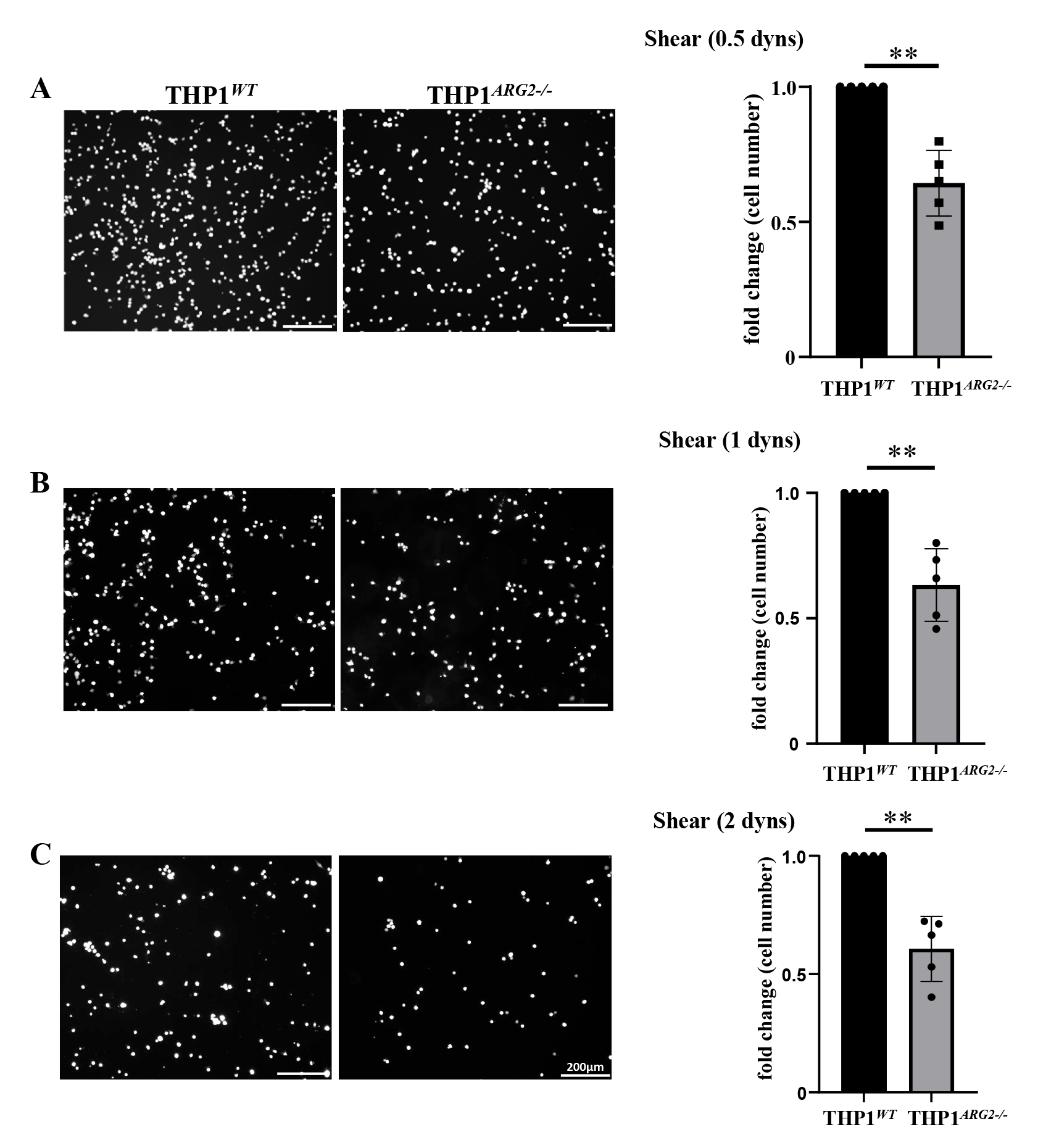

Supplement: Supplementary file 3 — Supplementary Material 3 [file 12929_2026_1278_MOESM3_ESM.tif]

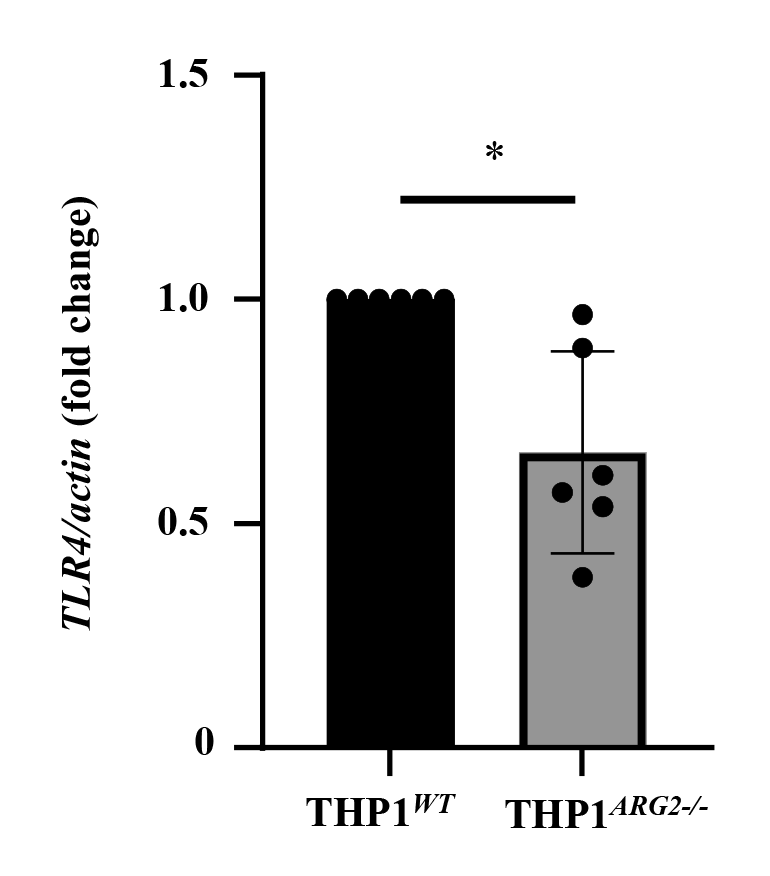

Supplement: Supplementary file 4 — Supplementary Material 4 [file 12929_2026_1278_MOESM4_ESM.tif]

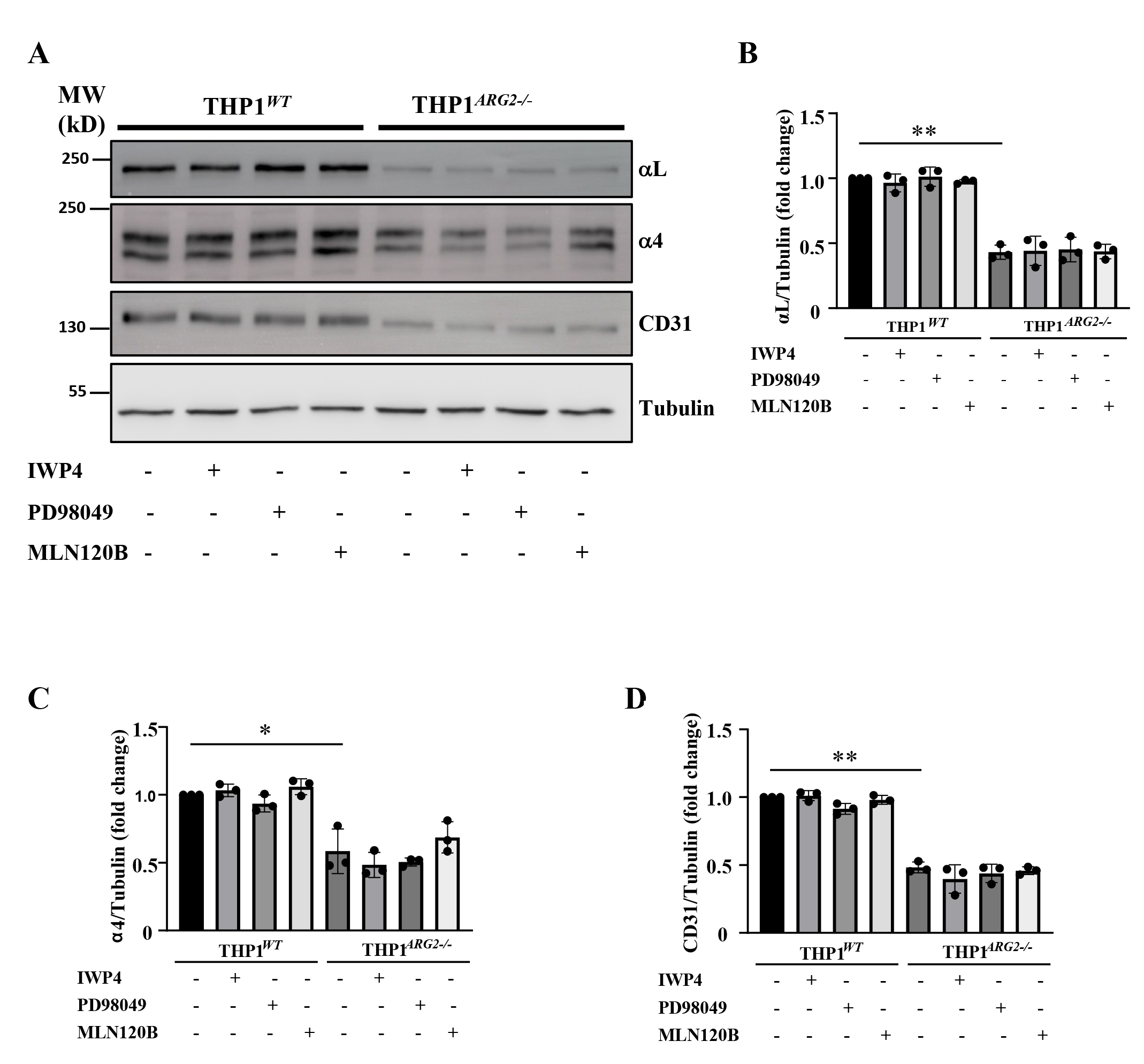

Supplement: Supplementary file 5 — Supplementary Material 5 [file 12929_2026_1278_MOESM5_ESM.tif]
